# PROTEOFORMER: deep proteome coverage through ribosome profiling and MS integration

**DOI:** 10.1093/nar/gku1283

**Published:** 2014-12-15

**Authors:** Jeroen Crappé, Elvis Ndah, Alexander Koch, Sandra Steyaert, Daria Gawron, Sarah De Keulenaer, Ellen De Meester, Tim De Meyer, Wim Van Criekinge, Petra Van Damme, Gerben Menschaert

**Affiliations:** 1 Lab of Bioinformatics and Computational Genomics, Department of Mathematical Modeling, Statistics and Bioinformatics, Faculty of Bioscience Engineering, Ghent University, Ghent, Belgium; 2 Department of Medical Protein Research, Flemish Institute of Biotechnology, Ghent, Belgium; 3 Department of Biochemistry, Faculty of Medicine and Health Sciences, Ghent University, Ghent, Belgium

## Abstract

An increasing amount of studies integrate mRNA sequencing data into MS-based proteomics to complement the translation product search space. However, several factors, including extensive regulation of mRNA translation and the need for three- or six-frame-translation, impede the use of mRNA-seq data for the construction of a protein sequence search database. With that in mind, we developed the PROTEOFORMER tool that automatically processes data of the recently developed ribosome profiling method (sequencing of ribosome-protected mRNA fragments), resulting in genome-wide visualization of ribosome occupancy. Our tool also includes a translation initiation site calling algorithm allowing the delineation of the open reading frames (ORFs) of all translation products. A complete protein synthesis-based sequence database can thus be compiled for mass spectrometry-based identification. This approach increases the overall protein identification rates with 3% and 11% (improved and new identifications) for human and mouse, respectively, and enables proteome-wide detection of 5′-extended proteoforms, upstream ORF translation and near-cognate translation start sites. The PROTEOFORMER tool is available as a stand-alone pipeline and has been implemented in the galaxy framework for ease of use.

## INTRODUCTION

The integration of next-generation transcriptome sequencing and highly sensitive mass spectrometry (MS) has emerged as a powerful strategy for the fast and comprehensive profiling of mammalian proteomes (1). Protein sequence database search tools (2) typically use publicly available protein databases, such as Swiss-Prot and Ensembl, to match MS spectra to peptides. Because these reference databases only contain experimentally verified and/or predicted protein sequences, it is very unlikely that they give a comprehensive assessment of the expressed protein pool of a given sample. Translation product prediction based on messenger RNA sequencing (mRNA-seq) data gives a more representative state of the protein repertoire expressed and aids the protein identification process by eliminating unexpressed gene products from the search space (3). On top of that, transcript data additionally provides sequence variation information, such as single nucleotide polymorphisms (SNPs) and RNA-splice and editing variants (4), which improve the chances of identifying novel protein forms (5,6).

Despite the benefits of adding mRNA-seq information to proteomics experiments, this approach has some shortcomings. First, mRNA levels are not a perfect proxy for protein expression levels since the translation of mRNA is subject to extensive regulation (7). Furthermore, there are several factors, such as internal ribosome entry sites, non-AUG start codons and non-sense read-through (8), that hinder the prediction of the exact protein product(s) translated from the transcript sequence. Also, inclusion of mRNA-seq information requires three- or six-frame-translation of the derived sequences, dramatically expanding the protein search space and hence decreasing the search sensitivity and specificity (9).

Recently a new strategy, termed ribosome profiling (RIBO-seq), was introduced that overcomes these shortcomings (8). By using the property of translating ribosomes to protect mRNA fragments from nuclease digestion it is possible to directly monitor the *in vivo* synthesis of mRNA-encoded translation products measured at the genome-wide level (10). In contrast to polysome profiling, often used for analyzing gene expression, RIBO-seq enables delineation of the genomic positions of translating ribosomes with subcodon to single-nucleotide precision (11). Furthermore, (alternative) translation initiation sites (TIS) can be accurately predicted by exploiting the abilities of antibiotics, such as harringtonine (HARR) or lactimidomycin (LTM), that halt ribosomes at sites of translation. However, as some non-coding transcripts show association with ribosomes (12), MS-assisted validation is in many cases still indispensable (13).

**Figure 1.**
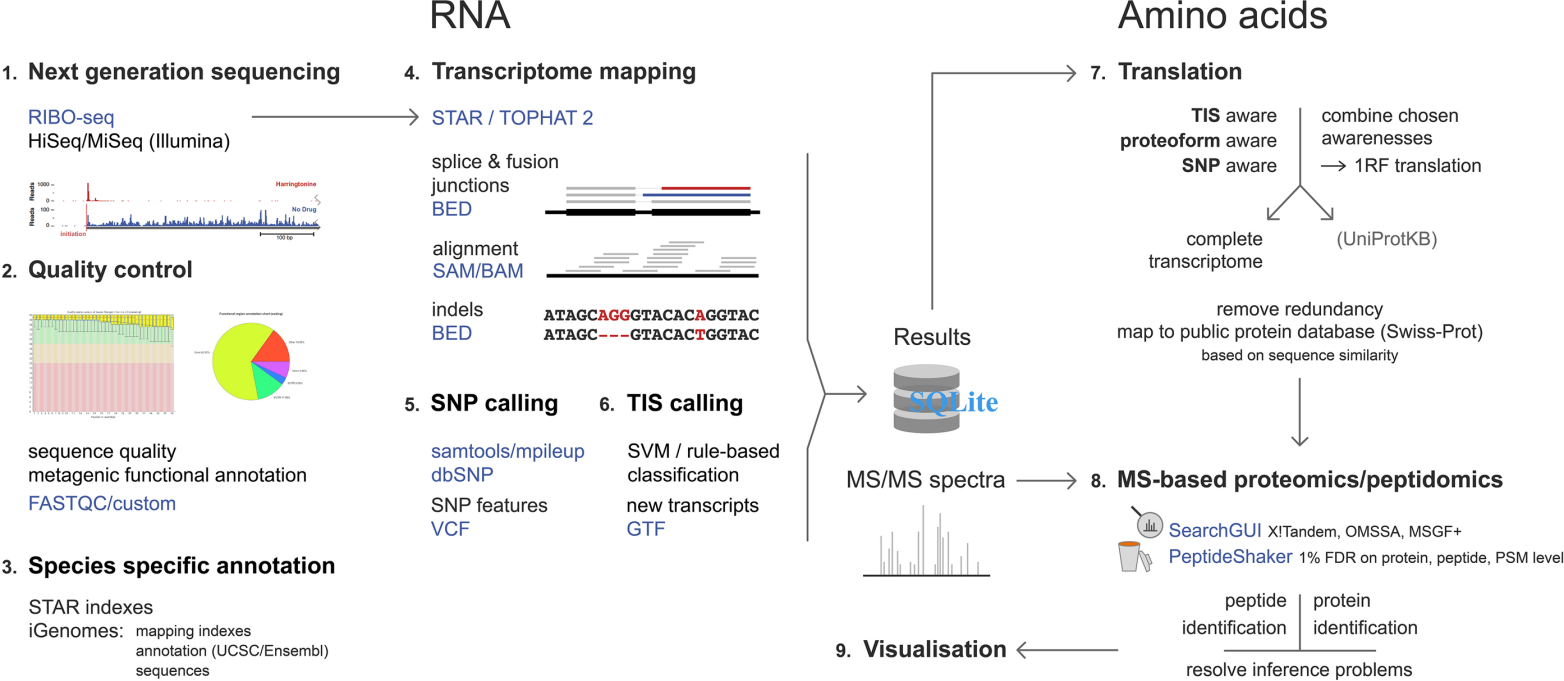
PROTEOFORMER integrates RIBO-seq and MS data. This schematic overview presents the different steps in the PROTEOFORMER workflow together with the used tools and file formats. The reads produced by a ribosome profiling experiment are first checked for their quality and subsequently mapped to the appropriate genome, using the STAR or TopHat transcriptome mapper and different species-specific annotation sources. Next, a SNP calling and a TIS prediction step are performed in order to accurately delineate the proteoforms. These RIBO-seq-derived proteoforms are then translated and mapped to a public protein database, creating a custom search space for an MS-based proteomics or peptidomics experiment. This protein sequence database created by PROTEOFORMER can then be used as a search space together with the SearchGUI and PeptideShaker tools (see Supplementary Methods S1) to identify proteoforms based on MS/MS spectra. The complete process of transforming RIBO-seq data into a custom search space is available as a stand-alone or Galaxy instance implementation.

The presented PROTEOFORMER tool processes RIBO-seq data allowing genome-wide visualization of protein synthesis, and moreover enables the delineation of *in vivo* proteoforms (14) building an optimal protein sequence search database for peptide to MS/MS matching (15–18) (Figure 1). PROTEOFORMER starts with the mapping of ribosome-protected fragments (RPFs) and quality control of subsequent alignments. It further includes modules for identification of transcripts undergoing protein synthesis, positions of translation initiation with subcodon specificity and SNPs. We used PROTEOFORMER to create protein sequence search databases from publicly available mouse (8) and in-house performed human RIBO-seq experiments and evaluated these with matching proteomics data. We demonstrate that this approach results in an increase of the number of protein/peptide identifications, leads to the identification of novel protein forms and aids in the re-annotation of the genome.

## MATERIALS AND METHODS

The PROTEOFORMER pipeline (Figure 1) is made up of six major steps: (i) the alignment of the RPF reads to a reference genome, (ii) a quality control of the alignments, (iii) assignment of transcripts with evidence of translation, (iv) identification of TIS, (v) inclusion of SNP information and (vi) finally generation of a RIBO-seq derived translation product database that can be used as a search space for MS-based proteomics studies, either independently or combined with a canonical protein database. All input parameters for the different steps of the PROTEOFORMER pipeline are user-definable in order to allow research-specific optimization. A more detailed description of the parameter settings is available via the readme file and website (Supplemental File S2 and http://www.biobix.be/proteoformer).

### Sequence processing and alignment

For the mouse and human sequences we use respectively the Ensembl (19) release 72 and 70 genome annotation (assembly GRCm38 and GRCh37) from the iGenome repository (http://support.illumina.com/sequencing/sequencing_software/igenome.ilmn).

RIBO-seq-derived reads can be aligned using both a STAR (20) (2.3.0e_r291) or TopHat (21) (v2.0.9) based pipeline. The STAR-based workflow sequentially aligns the reads to STAR indices composed of the following sequences: (i) the PhiX bacteriophage genome, (ii) *Mus musculus* or *Homo sapiens* rRNA (obtained using BioMart, filtered on Mt_rRNA and rRNA gene types) and (iii) *Mus musculus* or *Homo sapiens* complete genome (obtained from the corresponding iGenome repository). The STAR internal clipping function is used to clip the 3′ adaptor, up to two mismatches are allowed for the alignment, the option seedSearchStartLmaxOverLread is set to 0.5 and no introns are allowed for the alignment against the PhiX genome. The TopHat-based workflow uses Bowtie (v2.1.0) to sequentially align sequencing reads to Bowtie indices composed of the PhiX bacteriophage and the rRNA sequences (see above) using the ‘sensitive-local’ option, whereas TopHat itself is used for the complete genome alignment using default settings except for ‘segment-length’ that is set to 15. Since TopHat does not have an internal clipping functionality, the clipper from the FASTX Toolkit (0.0.13) is used to clip the 3′ adaptor sequence prior to mapping. For the RPF distribution plots and quality controls, only uniquely mapped reads are accounted for whereas for the custom DB creation multi-mapping reads (up to 15 locations) are additionally considered. Only reads with a length between 26 and 34 bases (i.e. relevant RPFs) are retained for further genomic coordinate mapping. RPF alignments are assigned to the current ribosomal P-site, based on the length of the fragment. The offset from the 5′-end of the alignment is +12, +13 and +14, respectively, for alignments ≤30 bases long, 31–33 bases long or ≥34 bases long (8). The alignment and RPF density information are returned as output by PROTEOFORMER (BedGraph format) making it easy to upload and visually evaluate the data in a genome browser environment of choice (22).

### Quality control: metagenic functional classification

As a first quality assessment, the obtained ribosomal footprints are classified using a species-specific Ensembl annotation bundle (converted to SQLite format). First, a metagenic functional annotation of the uniquely mapped footprints is determined using the Ensembl annotation of all transcripts. Here, translation associated annotation (i.e. 5′ untranslated region (UTR), exon, intron or 3′ UTR) is only defined for transcripts with a ‘protein-coding’ biotype. The RPFs not assigned to protein-coding transcripts are assigned to non-protein-coding transcripts (i.e. ‘other biotypes’). The remaining unassigned footprints are classified as ‘intergenic’. The resulting classification counts are available in a tab-separated table and summarized as a pie chart (Supplementary Figure S1a). For the ribosome footprints classified as ‘other biotypes’, a second table and accompanying pie chart is created, depicting the biotype distribution of these footprints (Supplementary Figure S1b).

### Gene distribution

The quality is also assessed by determining the uniquely mapped ribosomal footprint counts per gene (using available Ensembl annotation). In total, three summarizing plots are available: (i) a gene abundance plot ranging from the highest to the lowest covered genes, (ii) a cumulative gene distribution plot ranging form the highest to the lowest covered genes and (iii) a gene density plot (for more details, see Supplementary Figure S2). These results are also stored as tabular files.

### Transcript calling based on elongating ribosome coverage

Profiles of ribosomal footprints along a transcript are obtained by summing the number of footprints assigned to each genomic position of the coding sequence (CDS). The CDS of each known transcript is assembled using a species-specific Ensembl annotation bundle (converted to SQLite format). For non-protein-coding transcripts, the CDS is defined as the full exonic region of that specific transcript. For protein-coding transcripts, UTR-information is available, allowing us to determine the start and stop codons and to define the CDS as the exonic region between these two codons.

To remove variability in ribosomal footprint density due to RPF accumulation at start and stop codons (8,23), we additionally restrict the region where RPFs are counted by excluding the 15 nucleotides following each start codon and 15 nucleotides preceding each stop codon. For each transcript, the ribosomal footprint count is normalized based on the CDS length for which RPFs are taken into account (total CDS length – 30 bps). In order to identify the actual translated transcript isoforms, we examine the normalized footprint coverage of each of their exons. A transcript is denoted as truly translated if at least 85% of its exons have a coverage higher than or equal to a predetermined threshold. This transcript-specific threshold was set at an intuitive and robust value, namely, its mean exonic footprint coverage divided by 5. This excludes non-translated transcripts isoforms as well as allows (to some extent) possible variability in the ribosomal footprint density of real translated transcripts. Only transcripts that hold a fairly uniform footprint density throughout their CDS are subsequently classified as truly translated.

### TIS calling

The mapped profiles from the initiating ribosomes, obtained after harringtonine (HARR) or lactimidomycin (LTM) treatment, are accumulated at AUG or near-cognate start codons using a ±1 nt window, hence tackling the subcodon resolution issue (8,10). Profiles that do not map within this window relative to the first position of a start codon are disregarded during TIS calling. These accumulated peak positions have to comply with a number of criteria in order to be withheld as a true translation start site (10): (i) the identified TIS should have the maximal number of reads (HARR and/or LTM) within a window of 7 nucleotides (i.e. one codon up- and downstream), (ii) the combined number of ribosome profiles for the TIS should exceed a minimal profile count threshold and (iii) the TIS should have a $R_{LTM/HARR} - R_{CHX}$ value equal or higher than a certain threshold, where

\begin{equation*} R_k = \left( {X_k /N_k } \right) \times 10\quad (k = LTM\,or\,HARR,CHX) \end{equation*}

\begin{equation*} X_k = number\;of\;reads\;on\;position\;X\;for\;data\;k \end{equation*}

\begin{equation*} N_k = total\;number\;of\;reads\;on\;transcript\;for\;data\;k \end{equation*}

We opted for a categorized approach based on TIS localization; 5′ UTR, aTIS, CDS, 3′ UTR and no translation (TIS within non-protein-coding transcripts). aTIS that do not comply with the aforementioned criteria are also taken into account if the Ensembl transcript shows elongating ribosome occupancy. Hence, aTIS identifications are further divided in three subcategories; (i) those demonstrating accumulated TIS LTM/HARR coverage and compliant with all rules (TRUE), (ii) those having accumulated TIS LTM/HARR coverage, but not compliant with all rules (FALSE) and (iii) those without accumulated TIS LTM/HARR coverage (NO DATA). TIS in the other four categories that do not comply with these rules are discarded.

### SNP calling

Variants are extracted from the mapped RIBO-seq reads using SAMtools (24) (v.0.1.19) and by comparing the read mismatches to the NCBI dbSNP (25) data (build 137). The Picard toolkit (v.1.102: http://picard.sourceforge.net) is used to remove duplicates. Next, the variants are extracted using SAMtools mpileup coupled to BCFtools and the vcfutils.pl tool (both part of the SAMtools toolkit). To reduce the chances of missing variants with SAMtools, we also compare every mismatch in the mapped reads to the variants in dbSNP and any mismatch found in dbSNP is retained in the final set of variants. To keep the size of the search database manageable, the number of dbSNP-matched mismatches is calculated per transcript (based on Ensembl annotation release 72 and 70 for mouse and human, respectively) and whenever this number is higher than five, the mismatches in this transcript are removed from the final variant list.

### Translation assembly; PROTEOFORMER-DB construction and integration with a canonical protein database (e.g. Swiss-Prot)

Fast assembly of the translated sequences is made possible by a binary reading technique; fetching the CDS exon sequences from the corresponding chromosome sequence files (available from the iGenome repository). The aforementioned proteoform information (transcript isoform, TIS, SNP) is translated into the resulting amino acid sequence. Noteworthy is that only information on non-synonymous variations is presented in the translation product description. A custom, non-redundant translation product database for MS/MS-based protein/peptide identification is generated in FASTA format. The transcripts can be mapped to a known canonical protein database (e.g. Swiss-Prot) either by using the Biomart framework (26) (ID-based mapping) or by Basic Local Alignment Search Tool (BLAST) searching (sequence-based mapping).

Redundant sequences are eliminated based on the ranking of the annotations (in decreasing order of likeliness aTIS, 5′ UTR, CDS, 3′ UTR, no translation). If two or more transcript IDs have the same sequence, the transcript ID with the most plausible annotation is retained. If SNP information is included and two transcripts have the same annotation type and sequence, then the transcript with SNP information is retained. If two or more sequences satisfy all the constraints then one is chosen randomly. All subsequences (i.e. sequences completely contained in another sequence) are also eliminated from the database.

The ID-based mapping only considers those transcripts with annotation types aTIS or 5′ UTR transcripts, the other annotation types (CDS, 3′ UTR, no translation) are mapped by BLAST search. The ID-based mapping option simply maps a given Ensembl transcript ID to a corresponding canonical ID using the Biomart framework. If two or more transcripts have the same sequence then the transcript with an existing canonical ID (Biomart-mapped) is retained. If two or more transcripts have a Biomart mapping then one with a higher annotation ranking is retained. The transcripts without any pre-mapped ID could then be mapped by the sequence-based methods. In the sequence-based mapping, redundant transcripts are removed based on their annotation ranking and length. If two transcripts have the same sequence then the one with the most highly ranked annotation is retained and subsequently all subsequences are removed. The non-redundant sequences can then be mapped to known canonical proteins by performing a BLAST search against the canonical protein database (e.g. Swiss-Prot).

### PROTEOFORMER implementation

All information on the different implementations of the PROTEOFORMER method is available via http://www.biobix.be/proteoformer. A script-based (Perl 5) version and a Galaxy instance implementation are made available for download. These can respectively be deployed on a Unix system and implemented on a Galaxy instance (27). Furthermore, a customized virtual machine (Ubuntu 12.04 LTS) with all script dependencies and a Galaxy server already installed can be downloaded. A manual describing the aforementioned implementations (including prerequisites and dependencies) is made available on the website and as Supplementary Files S2 and S3.

### Supplementary methods

Additional information on the experimental procedures, MS data analysis and correlation analysis can be found in Supplementary Methods S1.

## RESULTS

In order to test the performance of the PROTEOFORMER method, we optimized different modules (mapping, TIS calling and SNP analysis) specifically toward the creation of a protein-synthesis based sequence database, using available mouse embryonic stem cell (mESC) RIBO-seq data (8). Matching shotgun and N-terminal COFRADIC (28) proteomics data served to evaluate this setup. While the former proteomics strategy gives a global assessment of the expressed proteome, the latter technique enables the isolation of N-terminal peptides, making it very appropriate for the validation of the by RIBO-seq observed (alternative) TIS.

### Optimization

Two different mapping tools (STAR (20) and TopHat2 (21)) were evaluated and both performed similarly in terms of the percentage of reads mapped onto the reference genome (Supplementary Table S2). However, STAR was selected for the rest of the analysis because it aligned slightly more relevant RPFs (i.e. with length between 26 and 34 bases), providing an increase of 2.85% and 4.6% for RPF of elongating and initiating ribosomes, respectively (Supplementary Figure S3). It also outperformed TopHat2 in terms of speed.

**Figure 2.**
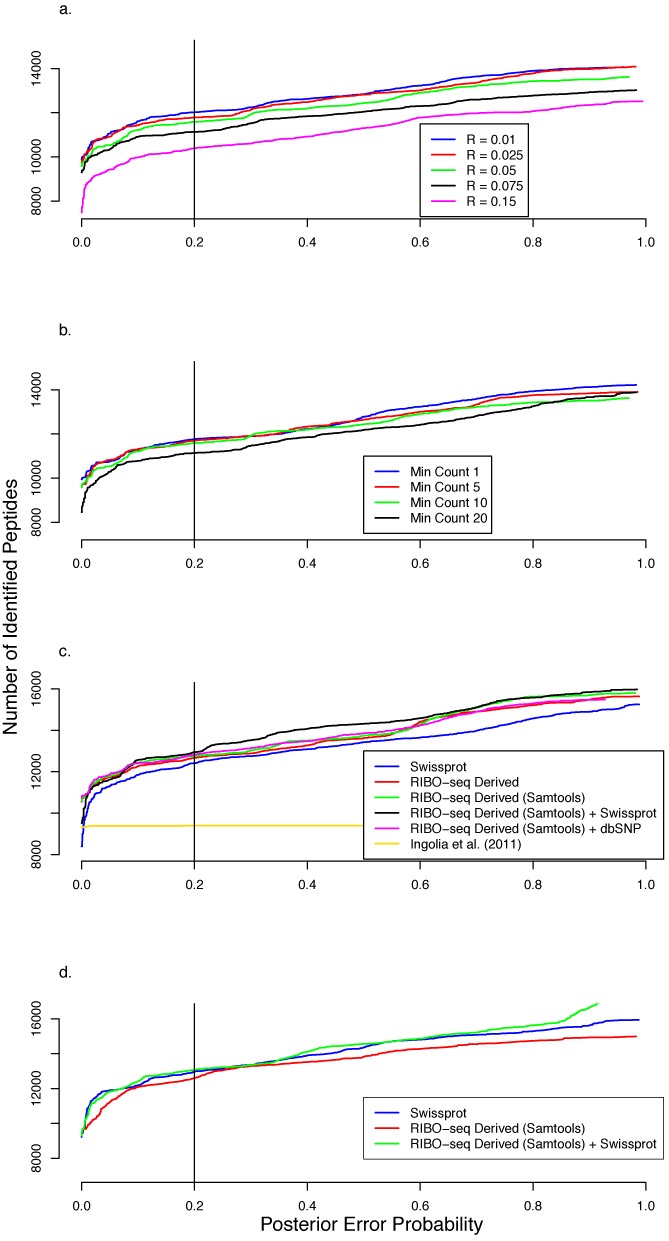
PEP distributions of the number of identified tryptic peptides from shotgun proteome analyses. The searches were performed on a database holding a non-redundant set of tryptic peptides based on the RIBO-seq-derived sequences having annotated TIS (aTIS). These plots demonstrate the impact of the database creation parameters of PROTEOFORMER on downstream MS/MS identification. The cumulative number of peptides identified is plotted on the *y*-axis and the corresponding PEP (i.e. the probability that a peptide-to-spectrum match is a chance event) is plotted on the *x*-axis. (a) mESC shotgun data: The effect of changing the *R*_LTM/HARR_ – *R*_CHX_ in the TIS calling procedure on the number of tryptic peptides identified with ‘minimum profile count’ (TIS calling) set to 10. The number of identified tryptic peptides decreases with increasing *R*_LTM/HARR_ – *R*_CHX_ value. There is a marked increase in the number of highly confident matches (for PEP < 0.2) at lower values of *R*_LTM/HARR_ – *R*_CHX_. (b) mESC shotgun data: The effect of ‘minimum profile count’ on the number of identified tryptic peptides at constant *R*_LTM/HARR_ – *R*_CHX_ of 0.01. The number of highly confident identifications decreases with increasing number of ‘minimum profile count’. At a confidence of about 80% (PEP < 0.2) the number of identified peptides is about the same for ‘minimum profile count’ 1 and 5. (c) mESC shotgun data: Comparison of the peptide identification numbers using different database versions. From the PEP distributions it is clear that searches using the RIBO-seq-derived database outperformed those using solely Swiss-Prot. With SNP information (RIBO-seq (SAMtools)) included, the number of identification increases even more, with the best result obtained using a search space combining RIBO-seq-derived sequences (SNP information inclusive) and Swiss-Prot entries at an 80% confidence validation threshold. It is also clear that the rule-based algorithm outperformed the SVM-algorithm applied in Ingolia *et al*. (8). (d) HCT116 shotgun data: The number of peptide identifications using only RIBO-seq-derived sequences as a search space is lower than searching Swiss-Prot. Yet a significant increase is notable when searching against a combined database (RIBO-seq derived + Swiss-Prot).

To optimize the PROTEOFORMER TIS calling algorithm for aTIS transcripts, we varied the two main TIS calling parameters: i.e. the minimum profile count (min count) and the difference in the normalized reads between the treated and untreated samples (*R*_LTM/HARR_ – *R*_CHX_). By varying the min count and *R*_LTM/HARR_ – *R*_CHX_ values we evaluated their impact on the downstream peptide identification rates on the mESC data. To do this we compiled non-redundant tryptic peptide search spaces for a range of different *R*_LTM/HARR_ – *R*_CHX_ (0.01–0.15) and min count (1–20) values and used these for spectral matching and database searching. The best TIS calling parameters were selected based on the number of confident tryptic peptides identified at a PEP (Posterior Error Probability) cutoff of 0.2 as this corresponds to an False Discovery Rate (FDR) of 1% (Figure 2a and b). With the min count set to 5 and the *R*_LTM/HARR_ – *R*_CHX_ values varying from 0.01 to 0.15, we observed that as the *R*_LTM/HARR_ – *R*_CHX_ value decreased the number of identified peptides increased and converged to a maximum. Below an *R*_LTM/HARR_ – *R*_CHX_ value of 0.01 the number of identifications started decreasing indicating that more noise was allowed into the data and that it became difficult for the peptide identification algorithm to clearly distinguish the good hits from the bad ones. This was also observed below a value of 5 when the min count varied from 1 to 20 while setting the *R*_LTM/HARR_ – *R*_CHX_ value fixed at a constant value of 0.01. For these reasons, a combination of *R*_LTM/HARR_ – *R*_CHX_ = 0.01 and min count = 5 was used for further analysis of aTIS transcripts. Furthermore, the rule-based TIS calling clearly outperformed a Support Vector Machine (SVM) algorithm (8) in compiling a comprehensive list of TISs in our setup (Figure 2c).

For other TIS categories, more stringent threshold settings were used in order to limit the amount of false-positive RIBO-seq-derived transcripts. This is especially important for downstream CDS TISs (using a rule-based TIS calling approach), as this region is very prone to false positives because of high ribosomal occupancy levels. However, excluding less stringent CDS TISs does not have a great impact on the final protein sequence database. During translation assembly, and in order to eliminate redundancy, the majority of CDS TIS-based transcripts are removed anyway (see Materials and Methods). Moreover, a more stringent approach for non-annotated TISs also ensures that TISs, and subsequent transcripts, that still pass parameter settings have a much greater chance to be true positives and are definitely worth further investigation. Thresholds for a TIS located in the 5′ UTR were set to 10 (min count) and 0.05 (*R*_LTM/HARR_ – *R*_CHX_); for a TIS located in the downstream CDS 15 and 0.15 were used; for a TIS within the 3′ UTR or a TIS within a non-protein-coding transcript these thresholds were set to 10 and 0.05.

With the optimal parameters identified we then generated varying non-redundant tryptic peptide databases based on inclusion of non-synonymous mutation information obtained from the RIBO-seq data using different strategies. These databases were compared alongside a tryptic peptide database generated from the mouse Swiss-Prot protein sequences. A search space built from the combination of RIBO-seq-derived sequences with mutation information derived from SAMtools and Swiss-Prot performed better than one derived from RIBO-seq, Swiss-Prot and mutation information from dbSNP (25) in terms of the number of tryptic peptide identifications (Figure 2c). This indicated that SAMtools is able to capture mutation information brought about by RIBO-seq, which is lacking in dbSNP. These settings also proved optimal in analyzing the human colorectal cancer cell line (HCT116) RIBO-seq data (Figure 2d).

### Evaluation on mESC and HCT116 cell line material

**Figure 3.**
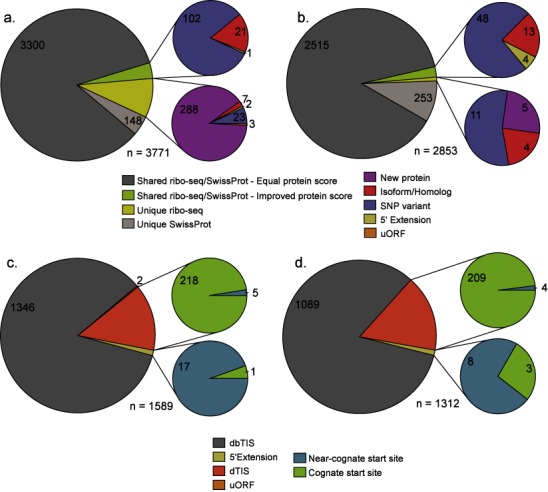
PROTEOFORMER enables deep proteome coverage. Pie charts representing the number of protein and peptide identifications obtained from the shotgun proteomics and N-terminal COFRADIC experiments based on searching the PROTEOFORMER + Swiss-Prot database for both mouse ESC cells and human HCT116 cells using a 1% FDR threshold. Execution times of the different modules used in order to arrive at these results can be found in Supplementary Table S3. (a) Shotgun proteomics results (mouse). A total of 3771 proteins were identified. (b) Shotgun proteomics results (human), identifying a total of 2853 proteins. For the shotgun experiments, a categorization was made based on the fact that the protein can be picked up using the PROTEOFORMER and/or Swiss-Prot sequence database. Also, the improved and new protein identifications were further classified into the following categories: new, isoform/homolog, SNP variant, 5′ extension and uORF. (c) N-terminal COFRADIC (mouse) experiment resulting in 1589 N-terminal peptide identifications. (d) N-terminal COFRADIC results (human). Here, 1312 N-termini were identified. The N-termini were categorized as either dbTIS (database annotated TIS), dTIS (downstream TIS), 5′ extension or uORF.

To evaluate the deep proteome coverage of the PROTEOFORMER pipeline, it was applied to the mESC and HCT116 RIBO-seq data sets. Combining the RIBO-seq-derived protein sequences with Swiss-Prot (mouse and human individually), 3771 mouse and 2853 human protein identifications were obtained from the shotgun experiments at a 1% FDR threshold (Figure 3a and b and Supplementary Table S1a and b). The supplemental (RIBO-seq-derived) sequences in the search space contributed to respectively 323 and 20 (8.6% and 0.7%) new and 124 and 65 (3.3% and 2.3%) improved protein identifications for the mouse and human data sets. These so-called new identifications were not contained in Swiss-Prot and originated from peptide identifications that (partly) overlapped an N-terminal extension, an exon region of an alternative spliced isoform, a mutation site or alternatively, an upstream open reading frame (uORF) (Figure 3a and b and Supplementary Figure S4a and b). Due to the increased protein coverage, these phenomena also accounted for a substantial increase of identifications with an improved protein score.

Correlation of the translational outcome based on ribosome profiling (RPF count) with the label-free protein abundance measures of the shotgun experiments (emPAI and NSAF) demonstrated that these technologies are highly complementary. Positive Pearson's correlation coefficients reaching up to 0.714 and 0.643 were obtained for mouse and human (18), respectively (Supplementary Figures S5 and S6), exceeding the correlation of the same MS spectral count-based measures with mRNA FPKM counts (1,6,29,30).

The N-terminal COFRADIC experiments resulted in the identification of different classes of N-termini (Figure 3c and d and Supplementary Table S1c and d). The majority of peptides mapped canonical start sites or Swiss-Prot database annotated TIS (dbTIS): 1346 mouse and 1089 human N-termini (i.e. 84.7% and 83.0% of all identified N-termini), 223 and 213 (14.0% and 16.2%) started downstream of the annotated TIS (dTIS; past protein position 2 in reference to Swiss-Prot). Interestingly, 18 and 11 peptides pointed to N-terminally extended proteoforms in mouse and human. Another two N-terminal peptides pointed to the translation of uORF (completely within the 5′ UTR or out-of-frame and overlapping with canonical CDS) for mouse. Moreover, analysis of N-terminal COFRADIC data using the PROTEOFORMER pipeline provided us with evidence of translation initiation at near-cognate start sites (non-AUG codons recoded to initiator methionines. Peptide-to-spectrum matches (PSMs) corresponding to peptides located in a uORF region were manually validated (Supplementary File S1) and possibly hint at true translation of these uORFs, although it cannot be ruled out that an unpredicted extended proteoform exists comprising this translated uORF sequence.

**Figure 4.**
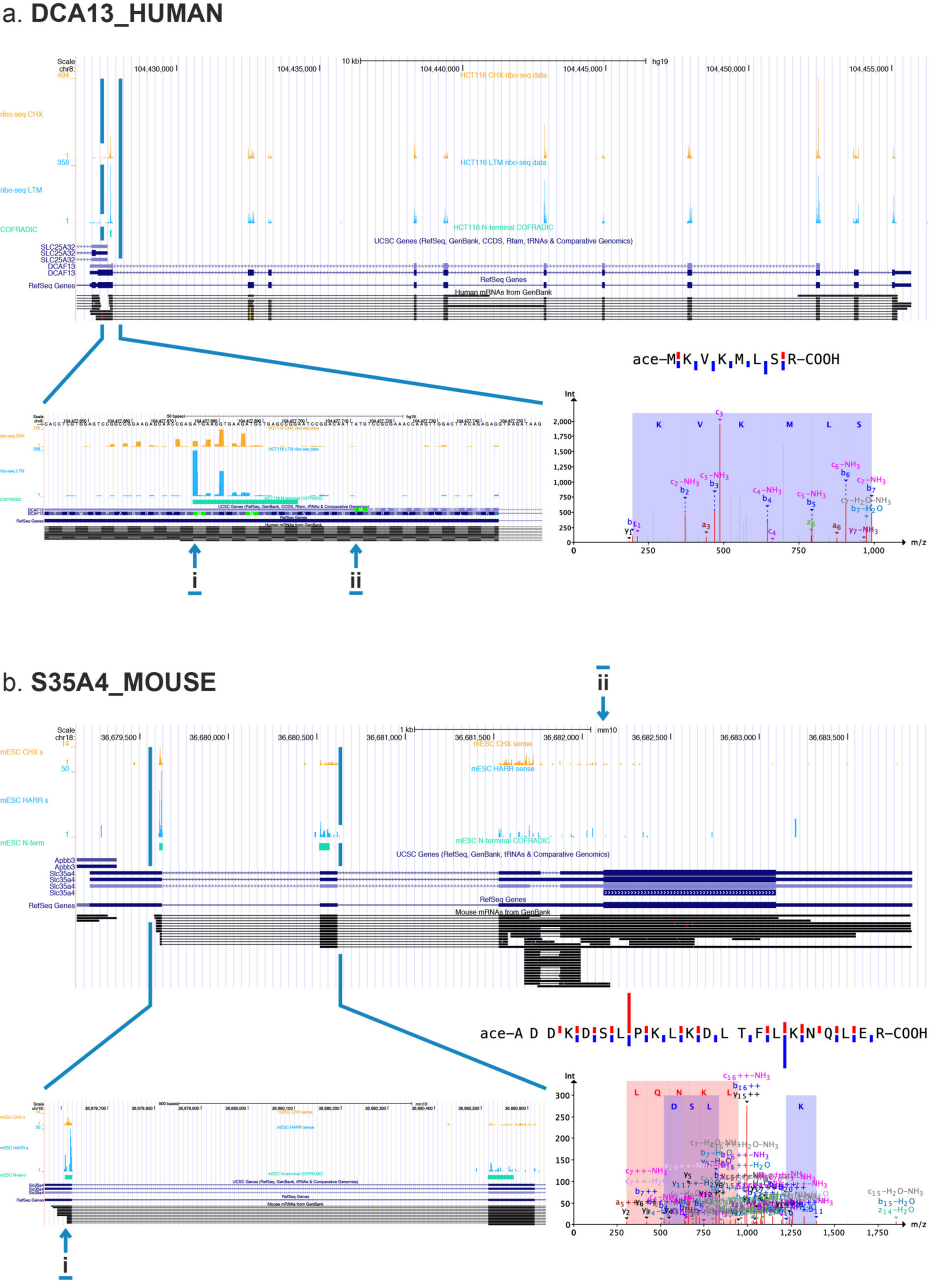
Examples of new proteoforms. (a) N-terminal extension of DCA13_HUMAN and (b) translated uORF of S35A4_MOUSE that were picked up by the proteogenomics analysis and validated by N-terminal COFRADIC. The UCSC genome browser was used to create a view of the RIBO-seq and COFRADIC data. The different tracks are from top to bottom: CHX-treated RIBO-seq data, LTM/HARR-treated RIBO-seq data, N-terminal COFRADIC data, UCSC genes, RefSeq genes and mRNA. The zoomed-in images show the alternative start site (i, alternative start site; ii, canonical start site), while the MS/MS spectra and sequence fragmentation plots display the confidence and quality of the N-terminal peptide identifications.

Interestingly, refined gene models can be built based on novel peptide identifications resulting from our PROTEOFORMER approach. These can be categorized into new exons (pointing to new isoforms, see Supplementary Table S1a and b), N-terminal extensions (see Supplementary Table S1 and examples of the human *dcaf13* and the orthologous *hdgf* gene illustrated in respectively Figure 4a and Supplementary Figure S7) and translation of uORFs (see the example of an uORF contained in the *Slc35a4* gene shown in Figure 4b). This uORF could also be categorized as a new gene product (resulting in a translation product of 103 AA, see Supplementary File S1). These findings suggest that the PROTEOFORMER approach can help to refine the annotation of the genome.

## DISCUSSION

PROTEOFORMER is the first publicly available analysis pipeline that provides a complete bioinformatics workflow for the analysis of RIBO-seq NGS data and that enables the construction of a customized protein sequence search space to allow integration with MS facilitating the capture of the proteome complexity. By combining the information from elongating and initiating ribosomes, it is able to create an optimal search space for matching MS experiments. The integration of PROTEOFORMER within the Galaxy framework provides a user-friendly interface for analysis of RIBO-seq data (in combination with proteomics data), resulting in new and improved identifications.

Noteworthy are the overall lower identification rates for the human sample. This can be attributed to (i) the fact that only heavy labeled peptides were considered in the human MS setup (Supplementary Methods S1), (ii) the overall better annotation of the human proteome (represented by the lower number of new non-Swiss-Prot identifications) and (iii) the higher number of identifications not present in our RIBO-seq-derived sequence pool (i.e. identifications matching Swiss-Prot entries only) for the human sample. Whereas only 148 (3.9%) identifications are not captured based the RIBO-seq strategy for the mouse data, this number increases to 253 (8.9%) for the human data. Inspection of the metagenic RPF abundance plots (Supplementary Figure S2) shows an expected dynamic range of expression. The quantitative correlation between RPF abundance and spectral count-based measures for the non-custom Swiss-Prot proteins (Supplementary Figure S8) demonstrates that this lower performance is not attributable to the CHX-treated HCT116 sample sequencing coverage. Finally, the distribution of the *R*_LTM/HAR_ – *R*_CHX_ values (used in the TIS calling procedure, see Materials and Methods and Supplementary Figure S9) pointed to an overall lower genome-wide coverage of initiating ribosomes, attributable to either biases introduced in the library preparation of the LTM-treated HCT116 sample or suboptimal conditions of the LTM treatment. Consequently, proteomics enables a quality assessment of RIBO-seq, which is typically lacking.

RIBO-seq-based studies also showed ribosome occupancy of long ncRNAs (lncRNAs) (8), possibly hinting toward their protein coding potential. However, most lncRNAs do not function through encoded proteins (31) demonstrating that RIBO-seq on its own is not a perfect proxy for protein synthesis and that MS validation is often indispensable (13). New RIBO-seq approaches as the Fragment Length Organization Similarity Score (32) and Ribosome Release Score (33) in combination with MS validation using, for example, PROTEOFORMER, will prove very useful in RIBO-seq-based protein identification (18).

By increasing the size of the sequence search space (e.g. a database derived from a six-frame translation of nucleotide sequences (based on mRNA-seq)), MS database search engines will underestimate the confidence assigned to the PSMs leading to fewer identifications at the estimated FDR and PEP thresholds (34) using a typical target-decoy approach. PROTEOFORMER only requires one-reading-frame translation in contrast to methods based on regular mRNA sequencing, thus limiting the search space explosion and keeping the confidence distribution of the search against the PROTEOFORMER database similar to standard Swiss-Prot searches (Figure 2c and d). We also envision that more efficient MS scoring algorithms (9) will be set in place to even better cope with the increasing search space sizes inherent to next-generation sequencing-based methods.

Through user-definable parameter settings, PROTEOFORMER provides the flexibility to tailor the creation of a translatome-based sequence database to the research question at hand. Downstream TIS identification or unbiased TIS calling are, for example, possible, but would need appropriate optimization for the different TIS categories. PROTEOFORMER makes use of iGenomes reference sequences and annotation from Ensembl for mapping, and custom Ensembl SQLite annotation databases (available on the PROTEOFORMER web page). It can already handle RIBO-seq-derived sequencing data of *Mus musculus, Homo sapiens, Drosophila melanogaster* and *Arabidopsis thaliana*, and we are currently working on incorporating other species. This is done on a case-by-case basis as species-specific adaptations, for example, to RPF parsing (23), are often desired. Furthermore, we are also continuously improving our pipeline including other TIS calling algorithms (8), SNP calling tools (35) and RIBO-seq specific measures (32,36).

In conclusion, we developed a new analysis pipeline, termed PROTEOFORMER. It enables the processing of RIBO-seq data and can be optimized based on user-definable parameter settings in order to be useful in answering a plethora of different research questions. The tool includes a mapping module enabling genome-wide visualization of ribosome occupancy on a genome browser of choice. It also includes a TIS calling algorithm that allows for the delineation of the ORFs of all translation products, based on initiating ribosome footprint accumulation obtained upon LTM/HARR treatment. A complete translatome-based sequence database, also including SNP information, can thus be compiled, for spectral database matching. We further showed that optimization toward the use of PROTEOFORMER in a proteogenomic approach, enables deep proteome coverage (including 5′ extended proteoforms, alternative spliced isoform and uORFs) resulting in an increase in overall protein identification rate when searching matching MS data sets.

A stand-alone version (Supplementary File S2) and a galaxy implementation (Supplementary File S3 and Supplementary Figure S10) of our approach are available at http://www.biobix.be/proteoformer next to all relevant information on the installation and underlying requirements.

## SUPPLEMENTARY DATA

Supplementary Data are available at NAR Online.

SUPPLEMENTARY DATA
