# Supplementary material for: PROTEOFORMER: deep proteome coverage through ribosome profiling and MS integration
Source: Nucleic Acids Res. 2014 Dec 15;43(5):e29. doi: 10.1093/nar/gku1283 (PMC4357689; doi:10.1093/nar/gku1283)
Supplement: SUPPLEMENTARY DATA [file supp_43_5_e29__index.html]

PROTEOFORMER: deep proteome coverage through ribosome profiling and MS integration — SUPPLEMENTARY DATA 

# PROTEOFORMER: deep proteome coverage through ribosome profiling and MS integration

## SUPPLEMENTARY DATA

**Files in this Data Supplement:**

- SUPPLEMENTARY DATA
- SUPPLEMENTARY DATA
- SUPPLEMENTARY DATA
- SUPPLEMENTARY DATA
- SUPPLEMENTARY DATA
- SUPPLEMENTARY DATA
