# Supplementary material for: PROTEOFORMER: deep proteome coverage through ribosome profiling and MS integration
Source: Nucleic Acids Res. 2014 Dec 15;43(5):e29. doi: 10.1093/nar/gku1283 (PMC4357689; doi:10.1093/nar/gku1283)
Supplement: SUPPLEMENTARY DATA [file supp_gku1283_nar-03263-met-g-2014-File006.pdf]

PROTEOFORMER: Deep Proteome Coverage through Ribosome Profiling and MS Integration

Supplementary Materials

Jeroen Crappé, Elvis Ndah, Alexander Koch, Sandra Steyaert, Daria Gawron, Sarah De Keulenaer, Ellen De Meester, Wim Van Criekinge, Petra Van Damme, Gerben Menschaert

Correspondence should be addressed to Gerben Menschaert

Lab of Bioinformatics and Computational Genomics (Biobix)
Ghent University
Coupure Links 653
9000 Ghent
Belgium

Email: gerben.menschaert@ugent.be
Phone: 0032/9 264 99 22

Supplementary Methods S1 - Experimental procedures, MS data and correlation analysis

Supplementary Figure S1 - Metagenic functional classification
Supplementary Figure S2 - Footpring gene distributions
Supplementary Figure S3 - RPF length distributions
Supplementary Figure S4 - Shotgun improved identification examples
Supplementary Figure S5 - RPF count correlation plots
Supplementary Figure S6 - RPF count correlation plots for validated aTIS transcripts
Supplementary Figure S7 - Depiction of the HDGF 5'-extension
Supplementary Figure S8 - RPF count correlation plots for Swiss-Prot unique proteins
Supplementary Figure S9 - $R_{LTM/HARR}$-R_{CHX} distribution for aTIS transcripts
Supplementary Figure S10 - PROTEOFORMER galaxy workflows

Supplementary File S1 - uORF manual curation
Supplementary File S2 - PROTEOFORMER script-based installation readme file
Supplementary File S3 - PROTEOFORMER galaxy implementation readme file

Supplementary Table S1 - General overview of peptide and protein identifications
Supplementary Table S2 - Mapping statistics
Supplementary Table S3 - Execution Time

Supplementary Methods S1

Cell culture for proteomics. For proteome analyses, E14Tg2a mESC cells (kindly provided by Prof. I. Chambers, University of Edinburgh) were cultivated as described previously (1). HCT116 cells (provided by the Johns Hopkins Sidney Kimmel Comprehensive Cancer Center, Baltimore, USA) were cultivated in DMEM medium supplemented with 10% fetal bovine serum (HyClone, Thermo Fisher Scientific Inc.), 100 units/ml penicillin (Gibco, Life Technologies) and 100 µg/ml streptomycin (Gibco) in a humidified incubator at 37°C and 5% CO_2. Prior to the proteomics experiments, the HCT116 cells were subjected to SILAC labeling (2) as part of another experiment that compares the wild type HCT116 cells to a double knockout line. For the N-terminal COFRADIC analysis, cells were transferred to media containing 140 µM heavy ($^{13}C_6{}^{15}N_4$) L-arginine (Cambridge Isotope Labs, Andover, MA, USA). For the shotgun proteome analysis, cells were cultured in medium supplemented with 140 µM medium heavy ($^{13}C_6$) L-arginine and 800 µM heavy ($^{13}C_6$) L-lysine. To achieve a complete incorporation of the labeled amino acids, cells were maintained in culture for at least 6 population doublings.

Cell culture and sample preparation for ribosome profiling. HCT116 cells were cultivated in McCoy's 5A (Modified) Medium (Gibco) supplemented with 10% fetal bovine serum, 2 mM alanyl-L-glutamine dipeptide (GlutaMAX, Gibco), 50 units/ml penicillin and 50 µg/ml steptamycin at 37°C and 5% CO_2. Cultures at 80-90% confluence were treated with 50 µM LTM or 100 µg/ml CHX (Sigma, USA) for 30 min at 37°C. Subsequently, cells were washed with PBS, harvested by trypsin-EDTA, suspended and washed again with PBS and recovered by 5 min of centrifugation at 1,500 × g, all in the presence of CHX to maintain the polysomal state. Cell pellets were resuspended in ice-cold lysis buffer, formulated according to Guo *et al.* (2010) (3) (10 mM Tris-HCl, pH 7.4, 5 mM $MgCl_2$, 100 mM KCl, 1% Triton X-100, 2 mM dithiothreitol (DTT), 100 mg/ml CHX, 1 × complete and EDTA-free protease inhibitor cocktail (Roche)), at a concentration of 40 × 10^6 cells/ml. After 10 min of incubation on ice with periodic agitation, lysed samples were passed through QIAshredder spin columns (Qiagen) to shear the DNA. Subsequently, the flow-throughs were centrifuged for 10 min at 16,000 × g and 4°C. The recovered supernatant was aliquoted, snap-frozen in liquid nitrogen and stored at -80°C for subsequent ribosome footprint recovery and cDNA library generation.

Shotgun proteome analysis. For shotgun proteome analyses, HCT116 and mESC E14 cells were lysed by 3 rounds of freeze-thaw lysis in 50 mM NH_4HCO_3 (pH 7.9). Lysates were cleared by centrifugation for 15 min at 16,000 g. Protein

concentrations were measured using the Protein Assay kit (Biorad) according to the manufacturer's instructions. To partially denature proteins, guanidinium hydrochloride (final concentration 0.5 M) and acetonitrile (final concentration 2%) were added to the cleared protein extracts. 1 mg of the protein sample was digested overnight at 37°C using sequencing-grade, modified trypsin (Promega, Madison, WI, USA) (enzyme/substrate of 1/100 w/w). Samples were acidified with acidic acid to a final concentration of 0.5%. The digest was vacuum dried and the equivalent of 500 µg of the original protein material was loaded onto a RP-HPLC column for fractionation as described previously (4). To prevent oxidation of methionines between RP-HPLC runs, methionines were oxidized in the injector compartment by transferring 20 µl of a freshly prepared aqueous 3% H_2O_2 solution to a vial containing 90 µl of the acidified peptide mixture (final concentration of 0.54% H_2O_2). This reaction proceeded for 30 min at 30°C. For chromatographic separation 100 µl peptide mixture was then immediately injected onto an RP-HPLC column (Zorbax® 300SB-C18 Narrow-bore, 2.1 mm internal diameter × 150 mm length, 5 µm particles, Agilent). Following 10 min of isocratic pumping with solvent A (10 mM ammonium acetate in water/ACN (98:2 v/v), pH 5.5), a gradient of 1% solvent B increase per minute (solvent B: 10 mM ammonium acetate in ACN/water (70:30 v/v), pH 5.5) was started. The column was then run at 100% solvent B for 5 min, switched to 100% solvent A and re-equilibrated for 20 min. The flow was kept constant at 80 µl/min using Agilent's 1100 series capillary pump with the 100 µl/min flow controller. Fractions of 0.5 min were collected from 20 to 80 min after sample injection (120 fractions). These peptide fractions were vacuum dried and fractions eluting 12 min apart were pooled by re-dissolving these in a final volume of 40 µl of 2 mM TCEP and 2% acetonitrile, similar to a pooling strategy described previously (4). In total, 24 samples were analyzed by LC-MS/MS.

N-terminal COFRADIC analysis. For N-terminal COFRADIC analyses, HCT116 and mESC E14 cells were lysed in 50 mM HEPES pH 7.4, 100 mM NaCl and 0.8% CHAPS containing a cocktail of protease inhibitors (Roche) for 10 min on ice and centrifuged for 15 min at 16,000 g at 4°C and the protein samples subjected to N-terminal COFRADIC as described by Staes *et al.* (2011) (5). To enable the assignment of *in vivo* Nt-acetylation events, all primary protein amines were blocked using a (stable isotopic encoded) N-hydroxysuccinimide ester at the protein level (i.e. NHS-$^{13}C_2D_3$-acetate) (6). Per proteome, 45 samples were analyzed by LC-MS/MS.

LC-MS/MS analysis. LC-MS/MS analysis was performed using an Ultimate 3000 RSLC nano LC-MS/MS system (Dionex, Amsterdam, The Netherlands) in-line connected to an LTQ Orbitrap Velos (Thermo Fisher Scientific, Bremen, Germany),

for shotgun samples, or a LTQ Orbitrap XL mass spectrometer (Thermo Fisher Scientific, Bremen, Germany), for N-terminal COFRADIC samples. 2 µl of the sample mixture was first loaded on a trapping column (made in-house, 100 µm internal diameter (I.D.) × 20 mm length, 5 µm Reprosil–Pur Basic-C18-HD beads, Dr. Maisch, Ammerbuch-Entringen, Germany). After back-flushing from the trapping column, the sample was loaded on a reverse-phase column (made in-house, 75 µm I.D. × 150 mm length, 3 µm C18 Reprosil–Pur Basic-C18-HD beads). Peptides were loaded with solvent A' (0.1% trifluoroacetic acid in 2% acetonitrile) and were separated with a linear gradient from 98% of solvent A'' (0.1% formic acid in 2% acetonitrile) to 50% of solvent B' (0.1% formic acid in 80% acetonitrile) with a linear gradient of 1.8% of solvent B' increase per minute at a flow rate of 300 nl/min, followed by a steep increase to 100% of solvent B'. The Orbitrap Velos and LTQ Orbitrap XL mass spectrometers were operated in data-dependent mode, automatically switching between MS and MS/MS acquisition for the ten or six most abundant peaks in a MS spectrum respectively. Mascot Generic Files were created from the MS/MS data in each LC run using the Distiller software (version 2.3.2.0).

Peptide/protein identification and interpretation. The protein and peptide searches were performed against our species-specific custom database with X! Tandem Sledgehammer (2013.09.01.1) and OMSSA 2.1.9 using the SearchGui (1.16.4) tool (7). For the shotgun proteome analyses, methionine oxidation to methionine-sulfoxide, pyroglutamate formation of N-terminal glutamine and acetylation of the N-terminus were selected as variable modifications. For the HCT116 samples, heavy labelled arginine ($^{13}C_6$) and lysine ($^{13}C_6$) were additionally selected as fixed modifications. Mass tolerance was set to 10 ppm on precursor ions and to 0.5 Da on fragment ions. The peptide charge was set to 2+, 3+, 4+. Trypsin was selected as the cleavage enzyme with one missed cleavage allowed. Cleavage was also allowed when arginine or lysine was followed by proline.

For the N-terminomics experiment, the generated MS/MS peak lists were searched with Mascot (version 2.3) (Mascot is compatible with the endoproteinase semi-Arg-C/P cleavage setting, see below). Mass tolerance on precursor ions was set to 10 ppm (with Mascot's C13 option set to 1) and to 0.5 Da on fragment ions. The peptide charge was set to 1+, 2+, 3+ and the instrument setting to ESI-TRAP. Methionine oxidation to methionine-sulfoxide, $^{13}C_2D_3$-acetylation on lysines and carbamidomethylation of cysteine were set as fixed modifications. Variable modifications were $^{13}C_2D_3$- acetylation, acetylation of peptide N-termini and pyroglutamate formation of N-terminal glutamine. For the HCT116 samples, $^{13}C_6{}^{15}N_4$ L-arg was additionally set as fixed modification. Endoproteinase semi-Arg-C/P (Arg-C specificity with arginine-proline cleavage allowed) was set as enzyme allowing for no missed cleavages.

Protein and peptide identification and data interpretation were done using the PeptideShaker algorithm (http://code.google.com/p/peptide-shaker, version 0.26.2), setting the FDR to 1% at all levels (peptide-to-spectrum matching, peptide and protein).

Ribosome profiling (RIBO-seq). The RIBO-seq of the HCT116 cells was executed as follows. 100 µl of the clarified HCT116 cell lysate (equivalent to 4×10^6 cells) was used as input for ribosome footprinting. The A260 absorbance of the lysate was measured with Nanodrop (Thermo Scientific) and for each A260, 5 units of ARTseq Nuclease (Epd icentre) were added to the samples. The nuclease digestion proceeded for 45 min at room temperature and was stopped by adding SUPERase.In Rnase Inhibitor (Life Technologies). Next, the ribosome protected fragments (RPFs) were isolated using Sephacryl S400 spin columns (GE Healthcare) according to the procedure described in 'ARTseq Ribosome Profiling Kit, Mammalian' (Epicentre). The RNA was extracted from the samples using acid 125 phenol:24 chloroform:1 isoamyl alcohol and precipitated overnight at -20°C by adding 2 µl glycogen, 1/10th volume of 5 M ammonium acetate and 1.5 volumes of 100% isopropyl alcohol. After centrifugation at 18,840 × g and 4°C for 20 min, the purified RNA pellet was resuspended in 10 µl nuclease free water.

Library preparation and sequencing. The HCT116 libraries were created according to the guidelines described in the ARTseq RIBO-seq Kit, Mammalian protocol (Epicentre). The RPFs were initially rRNA depleted using the Ribo-Zero Magnetic Kit (Human/Mouse/Rat, Epicentre), omitting the 50°C incubation step. Cleanup of the rRNA depletion reactions was performed through Zymo RNA Clean & Concentrator-5 kit (Zymo Research) using 200 µl binding buffer and 450 µl absolute ethanol. The samples were separated on a 15% urea-polyacrylamide gel and footprints of 26 to 34 nucleotides long were excised. RNA was extracted from the gel and precipitated. The pellet was resuspended in 20 µl nuclease-free water. Next, RPFs were end polished, 3' adaptor ligated, reverse transcribed and PAGE purified. 5 µl of circularized template DNA was used in the PCR reaction and amplification proceeded for 11 cycles. The libraries were purified with AMPure XP beads (Beckman Coulter) and their quality was assessed on a High Sensitivity DNA assay chip (Agilent technologies). The concentration of the libraries was measured with qPCR and they were single end sequenced on a Hiseq (Illumina) for 50 cycles.
Raw sequencing reads of the mESC RIBO-seq data (8) were downloaded from the Gene Expression Omnibus (dataset GSE30839). All reads from the control (cycloheximide treated, also referred to as CHX treated, sample GSM765292) and harringtonine treated (also referred to as HARR treated, sample GSM765295) were used.

Correlation analysis. Only the transcripts identified based on Swiss-Prot as well as our custom RIBO-seq derived translation products database were used for the correlation analysis. Quantification of ribosome occupancy was measured as ribosomal footprints per CDS (RPF count), hereby correcting for a possible 3'UTR and 5'UTR bias (8). Two quantitative measures for protein abundance based on spectral counts (emPAI (9) and NSAF (10)) were calculated using the shotgun proteomics data. While the first method (protein abundance index (PAI)) uses the number of peptides per protein normalized by the theoretical number of peptides, the NSAF method takes both the protein length and the total number of identified MS/MS spectra in an experiment into account. For each protein transcript with an aTIS for which quantitative RIBO-seq and shotgun proteomics information was available, a Pearson correlation coefficient was calculated between its normalized RPF count (based on CDS length) and its normalized spectral count. When more than one RIBO-seq-derived transcript corresponded to a particular Swiss-Prot protein sequence, the one with the highest normalized RPF count was used. The different normalization and identification approaches were combined with the following additional transcript filtering settings: *i)* no extra cutoffs, *ii)* only aTIS transcripts with a validated MS/MS-based identification (meaning that the spectral count value was ≥2), *iii)* only aTIS transcripts with a total RPF count ≥ 200 and *iv)* only aTIS transcripts with both a validated MS/MS-based identification and an RPF count ≥ 200. All correlation coefficients were computed using log-transformed RPF and emPAI/NSAF measures.

Data availability. All the MS data were converted using the PRIDE Converter(11) and are available through the PRIDE database (12) with the dataset identifier PXD000304 and DOI 10.6019/PXD000304 (for HCT116 MS experiments) and PXD000124 and DOI 10.6019/PXD000124 (for the mESC MS experiments).
The mESC datasets are publicly available, while the HCT116 datasets require a login (http://www.ebi.ac.uk/pride/archive/login, PX reviewer account: username: review48267, password: TTewpyNH).
The RIBO-seq libraries have been deposited in NCBI's Gene Expression Omnibus (13) and are accessible through the GEO series accession number GSE58207 (http://www.ncbi.nlm.nih.gov/geo/query/acc.cgi?acc=GSE58207).

Supplementary Figure S1

Mouse CHX

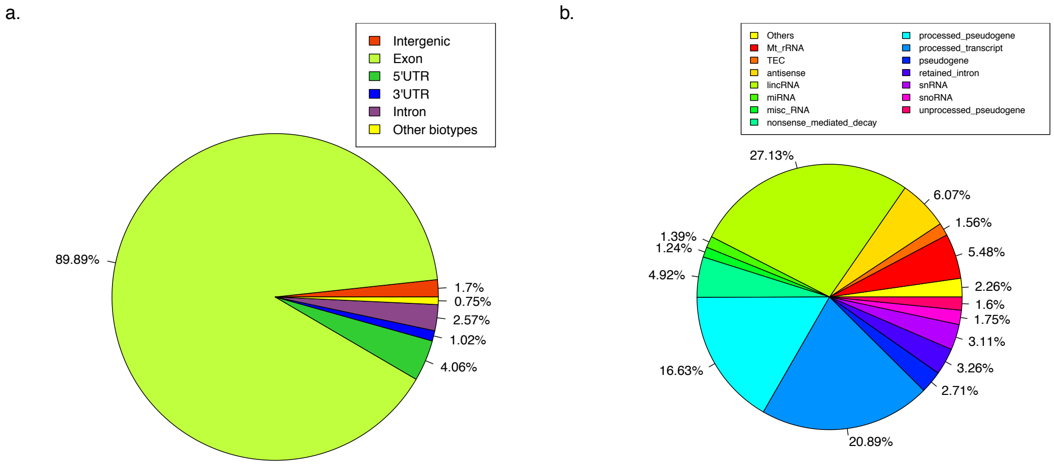

Mouse HARR

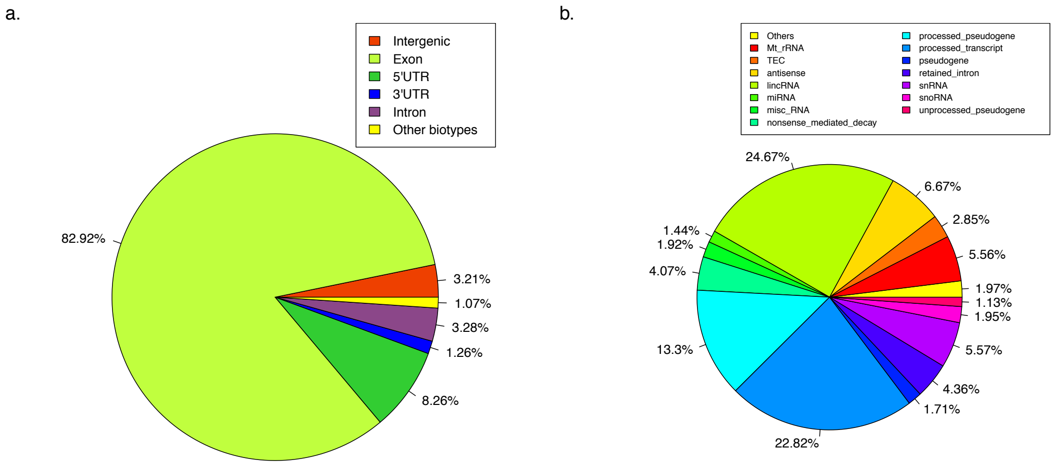

Human CHX

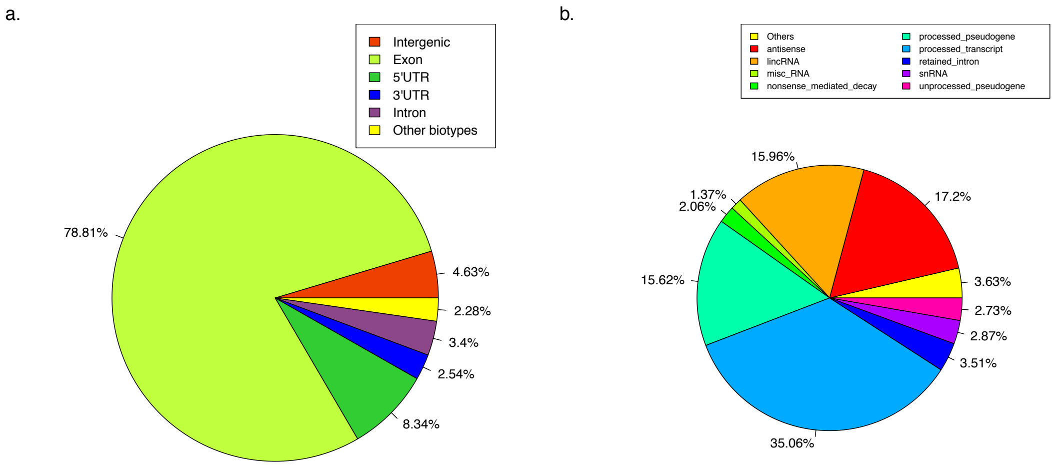

Human LTM

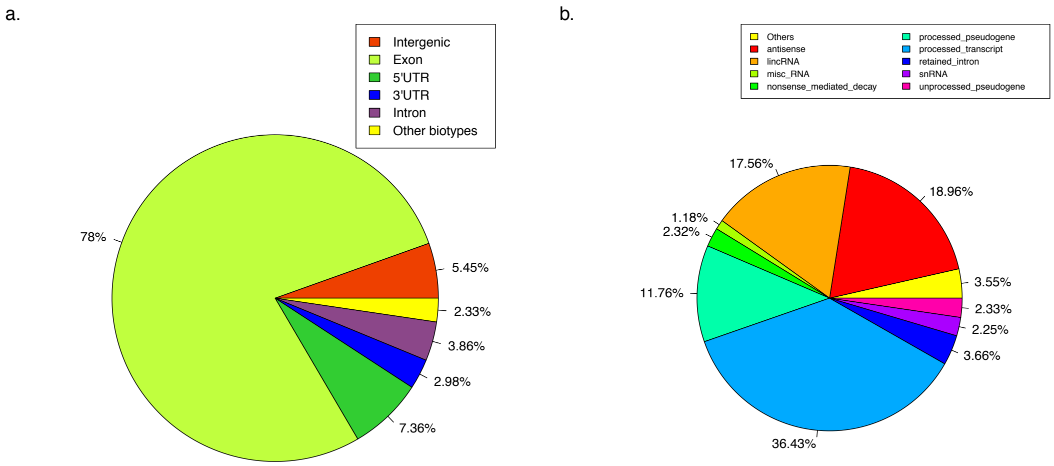

Supplementary Figure S1| Metagenic functional classification of the uniquely mapped RIBO-seq profiles deduced from the ribosome protected fragments (RPFs) of mouse and human elongating and initiating ribosomes. A first quality control classifies the obtained ribosome footprints using Ensembl gene annotations. **(a)** Pie chart representation of the percentage of RPFs that align to exonic, UTR and intronic regions of protein-coding transcripts. RPFs that could not be classified in one of these protein-coding transcripts, were assigned to non-protein-coding transcripts

(i.e. ‘other biotypes’) where possible, otherwise these are classified as intergenic. **(b)** Pie chart depicting the biotype distribution of the ribosome footprints classified as ‘other biotypes’ in chart (a).

Supplementary Figure S2

Mouse CHX

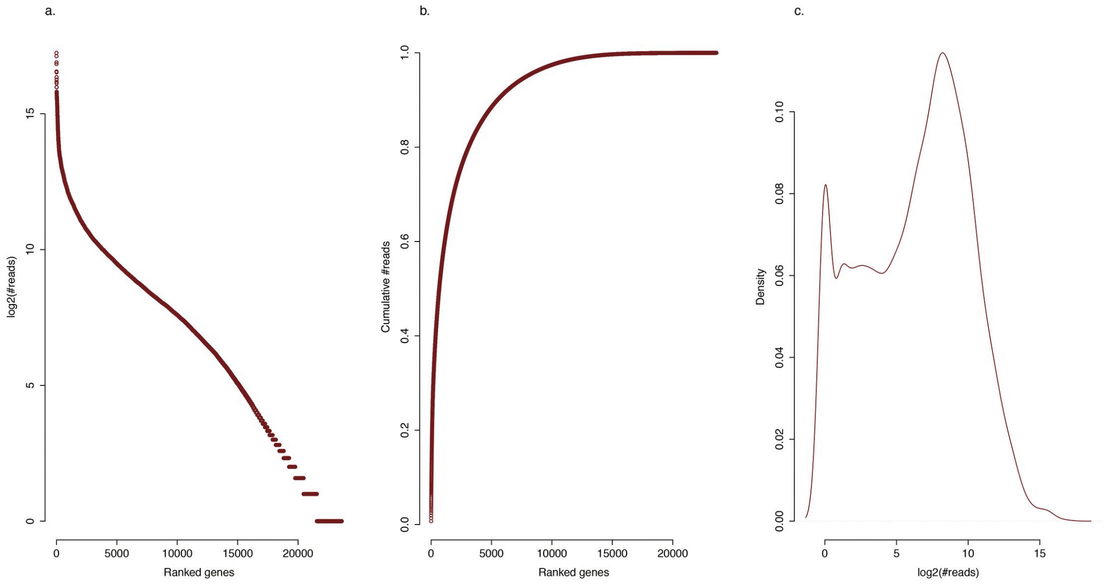

Mouse HARR

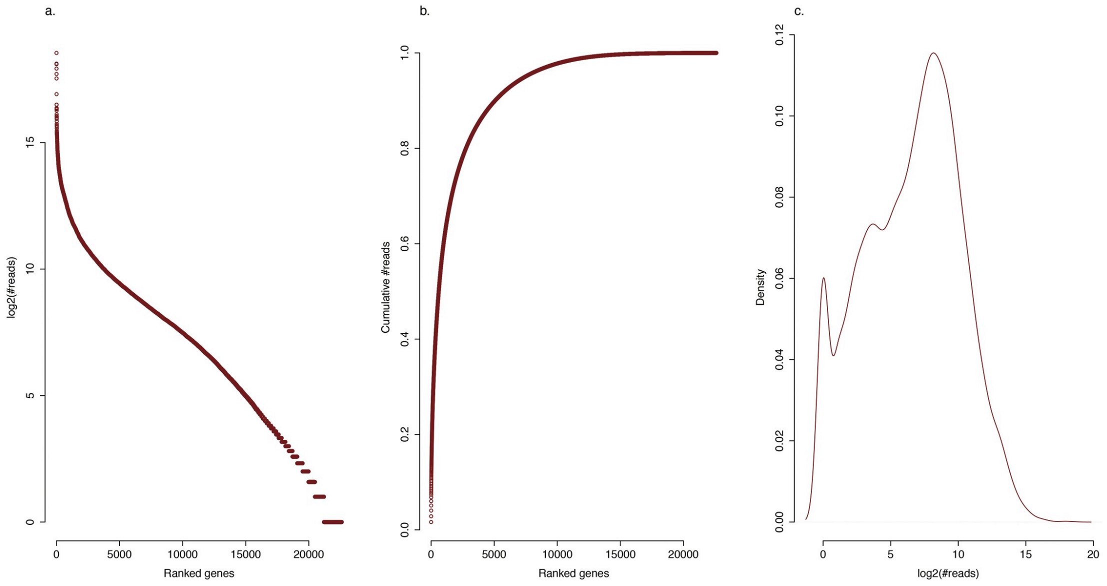

Human CHX

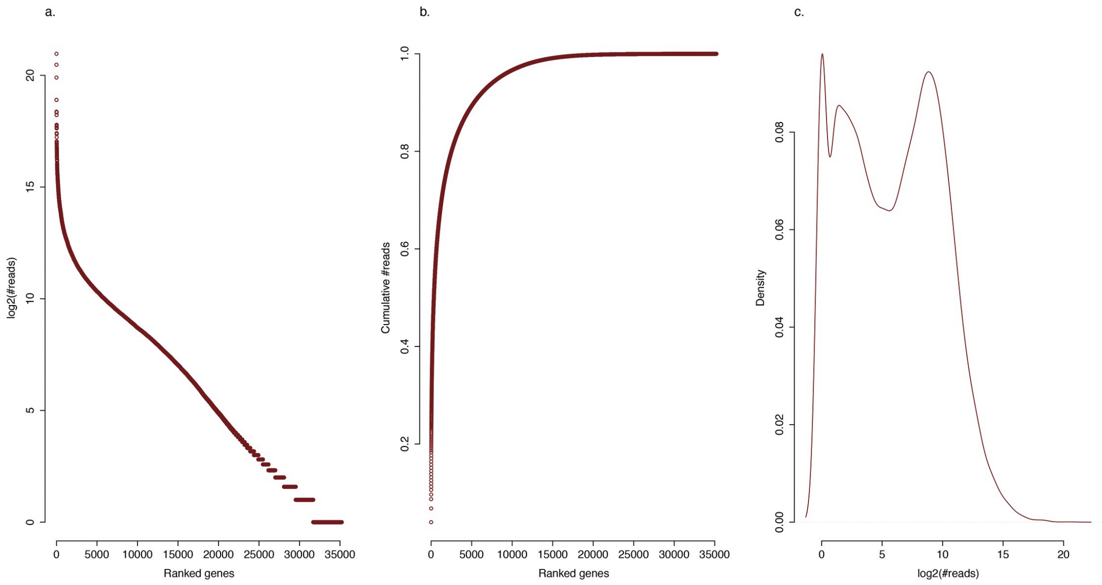

Human LTM

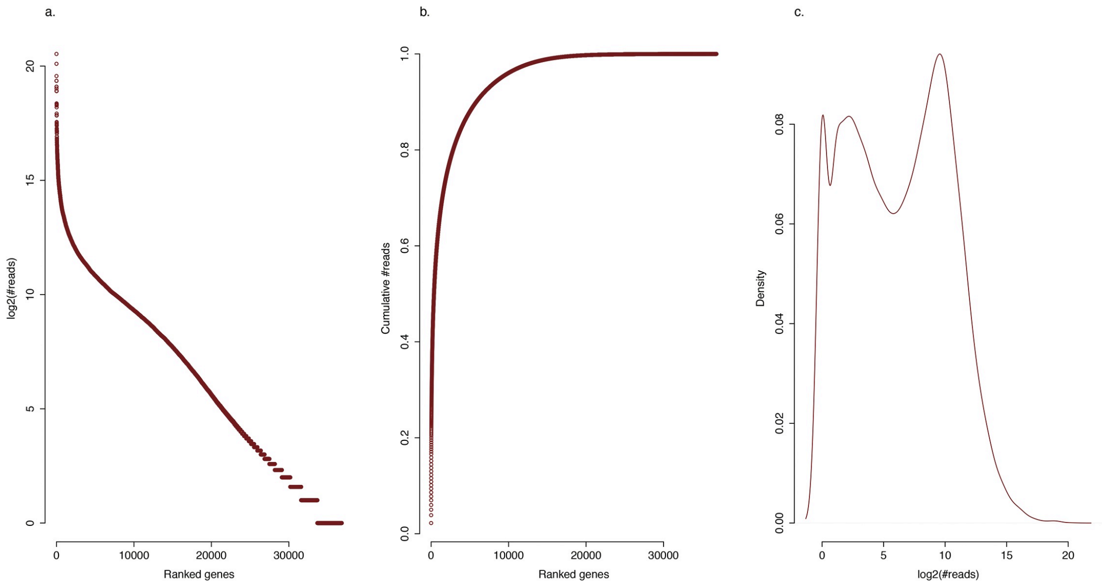

Supplementary Figure S2| Gene distributions of the ribosomal footprint count per gene for the uniquely mapped RIBO-seq profiles deduced from the RPFs of mouse and human elongating and initiating ribosomes. **(a)** Ranked gene abundance plot ranging from the most to the least covered genes. **(b)** Cumulative gene distribution plot ranging from the most to the least covered genes. **(c)** Gene density plot.

Supplementary Figure S3

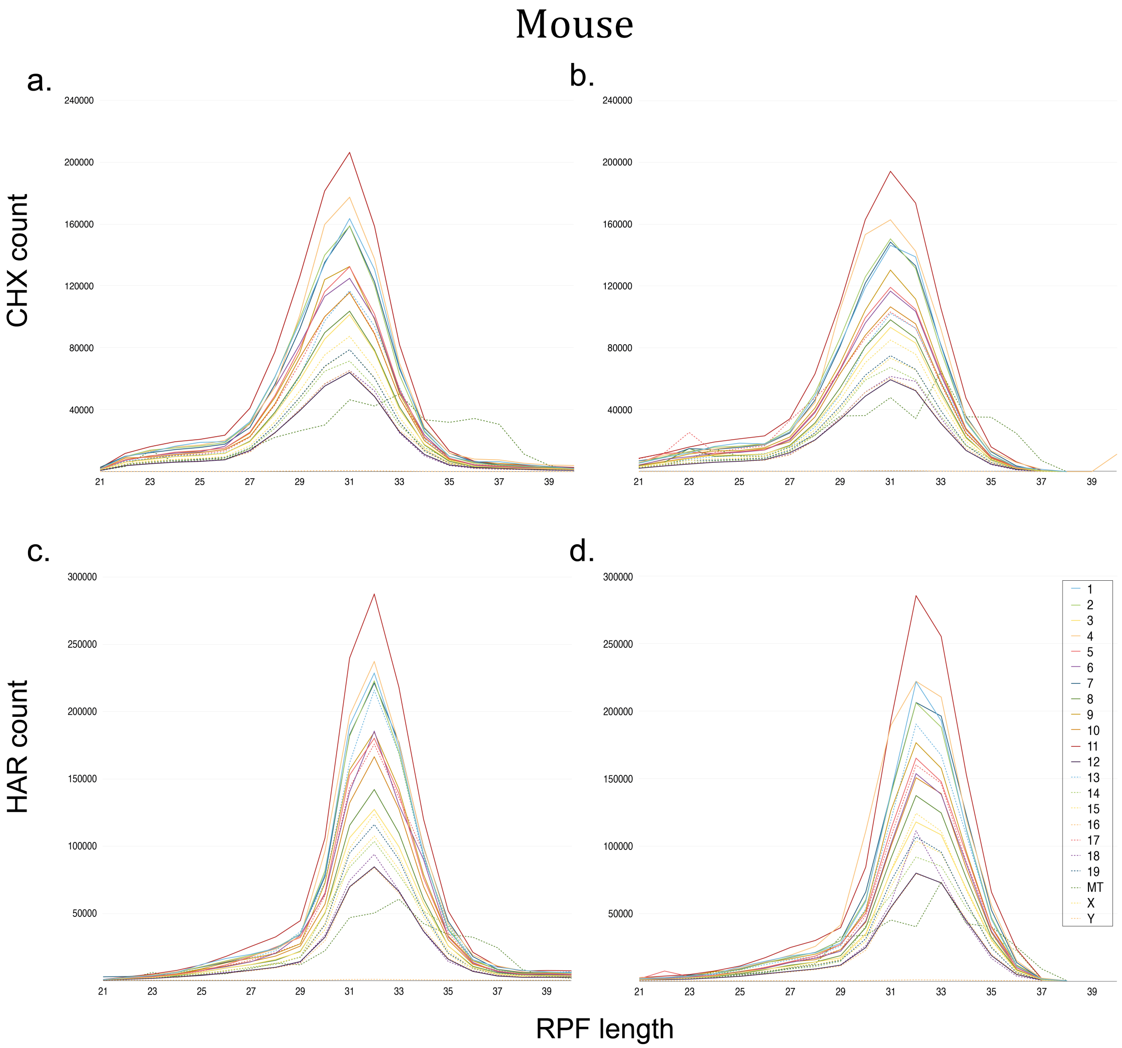

Human

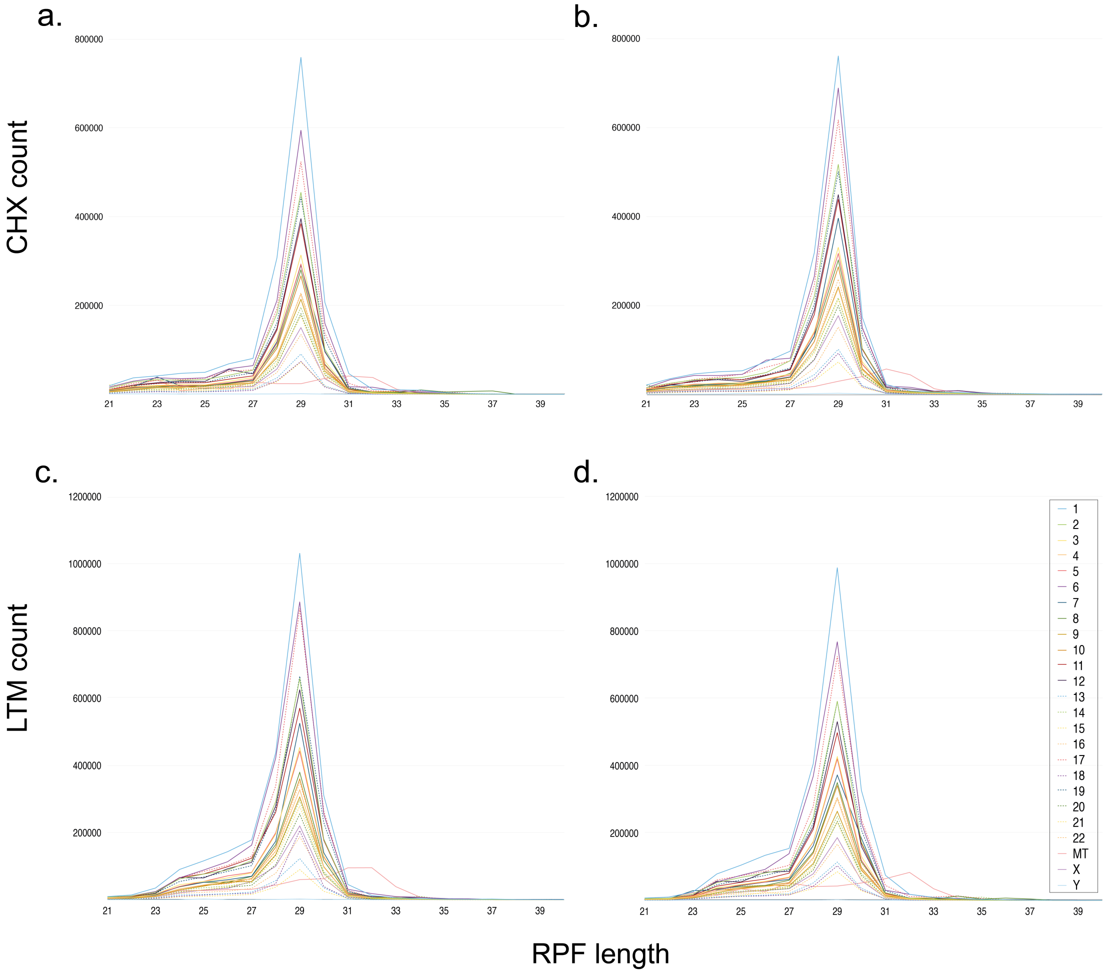

Supplementary Figure S3| RPF length distributions, split based on chromosomes, for mouse and human RIBO-seq data. **(a)** RPF length distribution of elongating ribosomes, based on STAR transcriptome mapper. **(b)** RPF length distribution of elongating ribosomes, based on TopHat transcriptome mapper. **(c)** RPF length distribution of initiating ribosomes, based on STAR transcriptome mapper. **(d)** RPF length distribution of initiating ribosomes, based on TopHat transcriptome mapper.

Supplementary Figure S4

A

5’-extension 1433E_MOUSE

```
sp|P62259|1433E_MOUSE                              --------------------------------MDDREDLVYQAKLAEQAERYDEMVESMK
generic|ENSMUST00000067664_11_75732932_5UTR|P62259 MFPARTRSEKLRESETLSASIRRADPAGAAAAMDDREDLVYQAKLAEQAERYDEMVESMK
                                                                                   ****************************

sp|P62259|1433E_MOUSE                              KVAGMDVELTVEERNLLSVAYKNVIGARRASWRIISSIEQKEENKGGEDKLKMIREYRQM
generic|ENSMUST00000067664_11_75732932_5UTR|P62259 KVAGMDVELTVEERNLLSVAYKNVIGARRASWRIISSIEQKEENKGGEDKLKMIREYRQM
                                                   ************************************************************

sp|P62259|1433E_MOUSE                              VETELKLICCDILDVLDKHLIPAANTGESKVFYYKMKGDYHRYLAEFATGNDRKEAAENS
generic|ENSMUST00000067664_11_75732932_5UTR|P62259 VETELKLICCDILDVLDKHLIPAANTGESKVFYYKMKGDYHRYLAEFATGNDRKEAAENS
                                                   ************************************************************

sp|P62259|1433E_MOUSE                              LVAYKAASDIAMTELPPTHPIRLGLALNFSVFYYEILNSPDRACRLAKAAFDDAIAELDT
generic|ENSMUST00000067664_11_75732932_5UTR|P62259 LVAYKAASDIAMTELPPTHPIRLGLALNFSVFYYEILNSPDRACRLAKAAFDDAIAELDT
                                                   ************************************************************

sp|P62259|1433E_MOUSE                              LSEESYKDSTLIMQLLRDNLTLWTSDMQGDGEEQNKEALQDVEDENQ
generic|ENSMUST00000067664_11_75732932_5UTR|P62259 LSEESYKDSTLIMQLLRDNLTLWTSDMQGDGEEQNKEALQDVEDENQ
                                                   ***********************************************
```

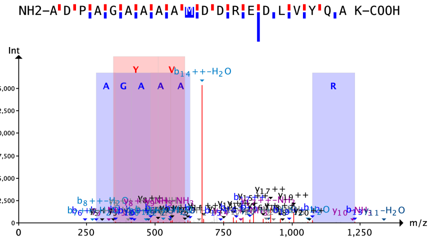

Mutation NASP_MOUSE

```
generic|ENSMUST00000030456_4_116627414_5UTR|Q99MD9 EIEAEIDPQVKSADVGGEEPKDQVATSESELGKAVLMELSGQDVEASPVVAAEAGAEVSE
sp|Q99MD9|NASP_MOUSE                               EIEAEIDPQVKSADVGGEEPKDQVATSESELGKAVLMELSGQDVEASPVVAAEAGAEVSE
                                                   ************************************************************

generic|ENSMUST00000030456_4_116627414_5UTR|Q99MD9 KPGQEITVIPNNGPVVGQSTVGDQTPSEPQTSAERLTETKDGSSVEEVKAELVPEQEEAM
sp|Q99MD9|NASP_MOUSE                               KPGQEITVIPNNGPVVGQSTVGDQTPSEPQTSAERLTETKDGSSVEEVKAELVPEQEEAM
                                                   ************************************************************

generic|ENSMUST00000030456_4_116627414_5UTR|Q99MD9 LPVEESEAAGDGVETKVAQRATEKAPEDKFKIAANEETQERDEQMKEGEETEGSEEEDRE
sp|Q99MD9|NASP_MOUSE                               LPVEESEAAGDGVETKVAQKATEKAPEDKFKIAANEETPERDEQMKEGEETEGSEEEDRE
                                                   *******************:****************** *********************

generic|ENSMUST00000030456_4_116627414_5UTR|Q99MD9 NDKAEETPNESVLEKKSLQENEEEEIGNLELAWDMLDLAKIIFKRQETKEAQLYAAQAHL
sp|Q99MD9|NASP_MOUSE                               NDKAEETPNESVLEKKSLQENEEEEIGNLELAWDMLDLAKIIFKRQETKEAQLYAAQAHL
                                                   ************************************************************

generic|ENSMUST00000030456_4_116627414_5UTR|Q99MD9 KLGEVSVESENYIQAVEEFQACLSLQEQYLEAHDRLLAETHYQLGLAYGYNSQYDEAVAQ
sp|Q99MD9|NASP_MOUSE                               KLGEVSVESENYIQAVEEFQACLSLQEQYLEAHDRLLAETHYQLGLAYGYNSQYDEAVAQ
                                                   ************************************************************
```

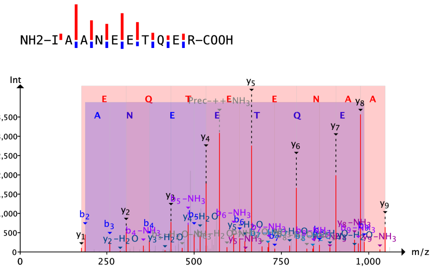

Isoform DNM1L_MOUSE

```
sp|Q8K1M6-5|DNM1L_MOUSE                            MEALIPVINKLQDVFNTVGADIIQLPQIVVVGTQSSGKSSVLESLVGRDLLPRGTGVVTR
generic|ENSMUST00000023477_16_16358865_aTIS|Q8K1M6 MEALIPVINKLQDVFNTVGADIIQLPQIVVVGTQSSGKSSVLESLVGRDLLPRGTGVVTR
sp|Q8K1M6-3|DNM1L_MOUSE                            MEALIPVINKLQDVFNTVGADIIQLPQIVVVGTQSSGKSSVLESLVGRDLLPRGTGVVTR
sp|Q8K1M6|DNM1L_MOUSE                              MEALIPVINKLQDVFNTVGADIIQLPQIVVVGTQSSGKSSVLESLVGRDLLPRGTGVVTR
sp|Q8K1M6-4|DNM1L_MOUSE                            ------------------------------------------------------------
sp|Q8K1M6-2|DNM1L_MOUSE                            MEALIPVINKLQDVFNTVGADIIQLPQIVVVGTQSSGKSSVLESLVGRDLLPRGTGVVTR

sp|Q8K1M6-5|DNM1L_MOUSE                            RPLILQLVHVSPEDKRKTTGEENG------VEAEEWGKFLHTKNKLYTDFDEIRQEIENE
generic|ENSMUST00000023477_16_16358865_aTIS|Q8K1M6 RPLILQLVHVSPEDKRKTTGEENG------VEAEEWGKFLHTKNKLYTDFDEIRQEIENE
sp|Q8K1M6-3|DNM1L_MOUSE                            RPLILQLVHVSPEDKRKTTGEENG------VEAEEWGKFLHTKNKLYTDFDEIRQEIENE
sp|Q8K1M6|DNM1L_MOUSE                              RPLILQLVHVSPEDKRKTTGEENGKFQSWRVEAEEWGKFLHTKNKLYTDFDEIRQEIENE
sp|Q8K1M6-4|DNM1L_MOUSE                            --------------------------------------------MLYTDFDEIRQEIENE
sp|Q8K1M6-2|DNM1L_MOUSE                            RPLILQLVHVSPEDKRKTTGEENGKFQSWRVEAEEWGKFLHTKNKLYTDFDEIRQEIENE
                                                                                                ***************

sp|Q8K1M6-5|DNM1L_MOUSE                            TERISGNNKGVSPEPIHLKVFSPNVVNLTLVDLPGMTKVPVGDQPKDIELQIRELILRFI
generic|ENSMUST00000023477_16_16358865_aTIS|Q8K1M6 TERISGNNKGVSPEPIHLKVFSPNVVNLTLVDLPGMTKVPVGDQPKDIELQIRELILRFI
sp|Q8K1M6-3|DNM1L_MOUSE                            TERISGNNKGVSPEPIHLKVFSPNVVNLTLVDLPGMTKVPVGDQPKDIELQIRELILRFI
sp|Q8K1M6|DNM1L_MOUSE                              TERISGNNKGVSPEPIHLKVFSPNVVNLTLVDLPGMTKVPVGDQPKDIELQIRELILRFI
sp|Q8K1M6-4|DNM1L_MOUSE                            TERISGNNKGVSPEPIHLKVFSPNVVNLTLVDLPGMTKVPVGDQPKDIELQIRELILRFI
sp|Q8K1M6-2|DNM1L_MOUSE                            TERISGNNKGVSPEPIHLKVFSPNVVNLTLVDLPGMTKVPVGDQPKDIELQIRELILRFI
                                                   ************************************************************
```

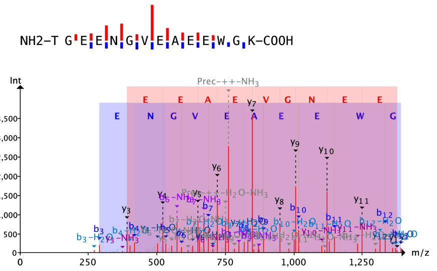

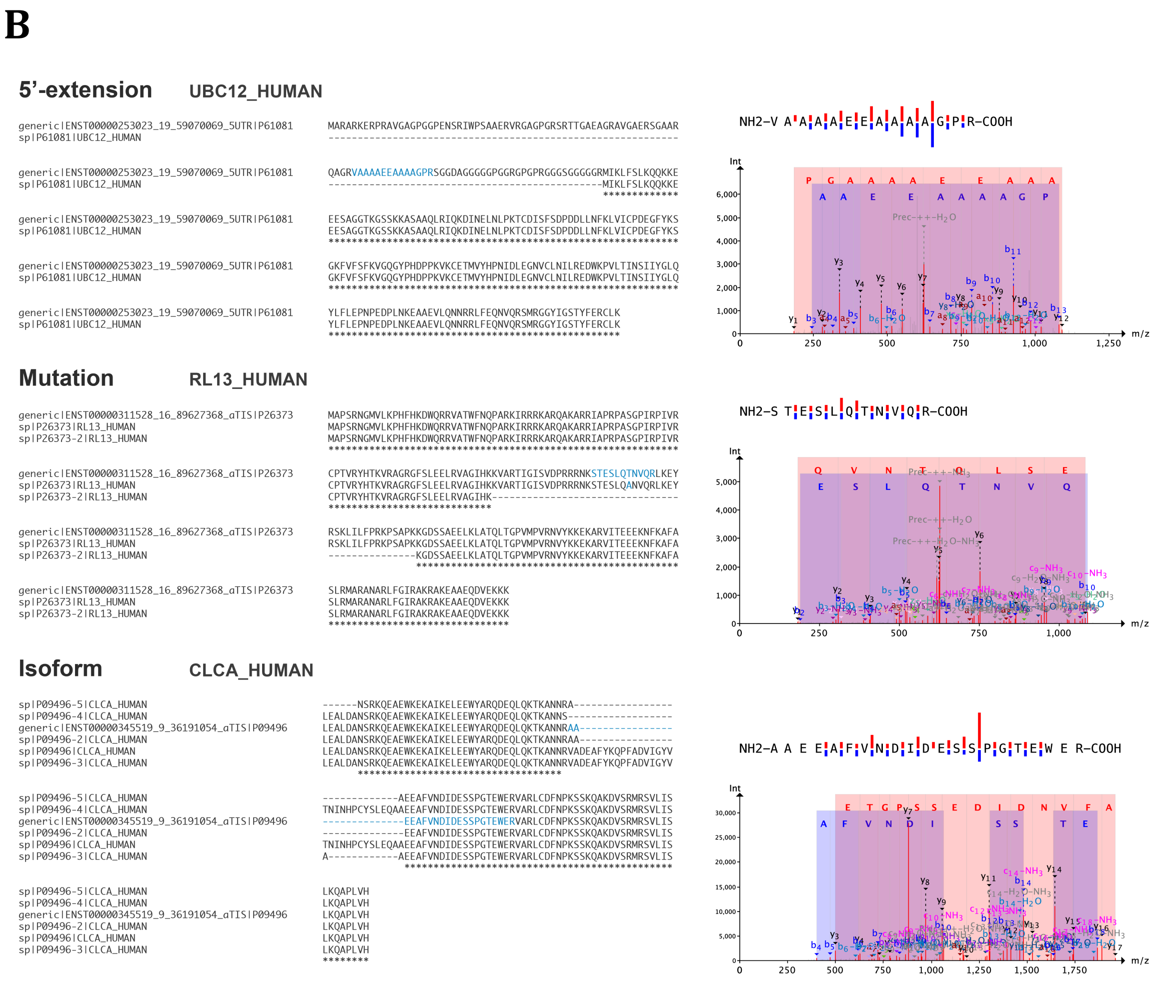

Supplementary Figure S4| Examples of improved identifications in the shotgun proteomics experiments. **(a)** The addition of RIBO-seq data to the mouse (mESC cells) proteomics experiment improved the identification and score significance for 124 proteins (See also **Supplementary Table S1**) and three representative examples are depicted here. The left column shows the Clustal Omega alignment of the RIBO-seq-derived amino acid sequences to the Swiss-Prot sequences with the relevant peptide identifications highlighted in blue. The column on the right shows the corresponding fragmentation spectra and peptide sequence fragmentations. **(b)** The addition of RIBO-seq derived translation products to the human (HCT116 cells) proteomics experiment improved the identification and score significance for 65 proteins of which three representative examples are depicted.

Supplementary Figure S5

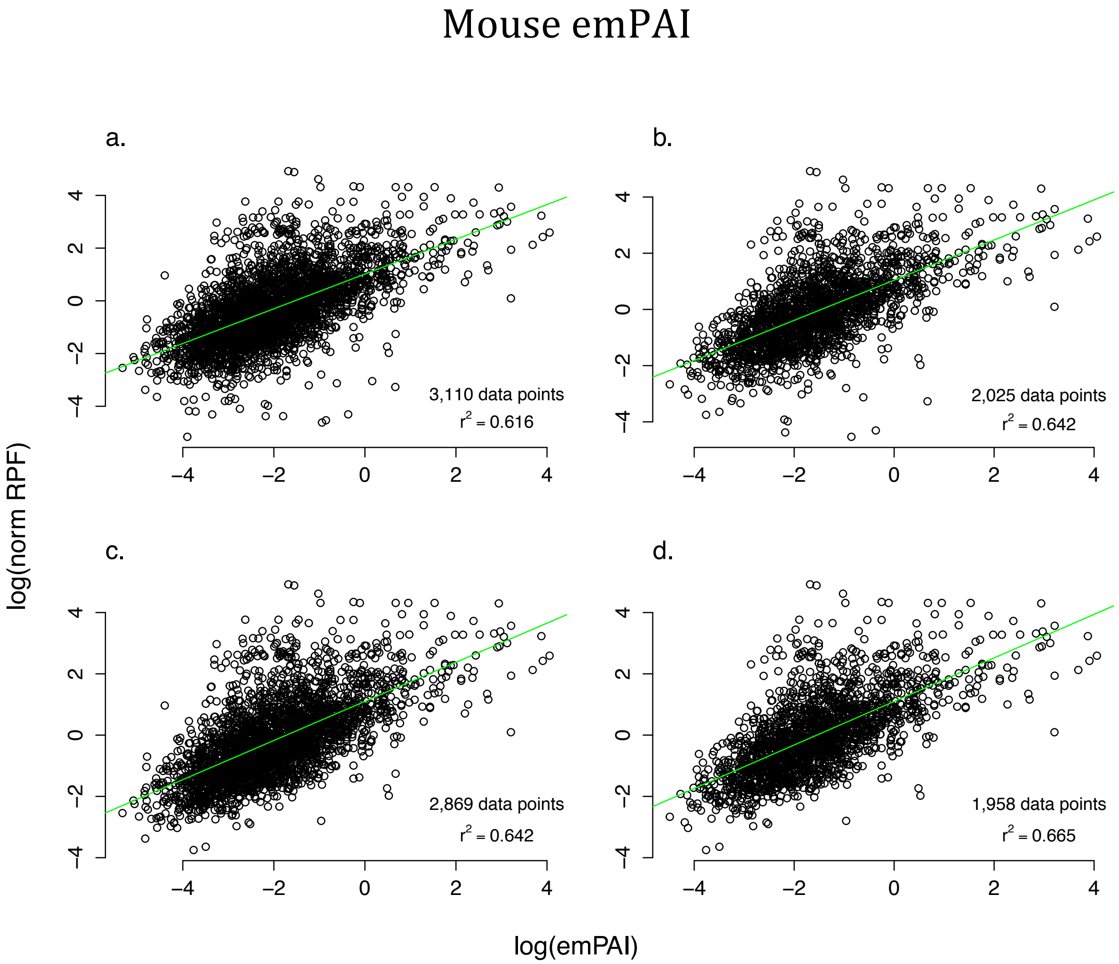

Mouse NSAF

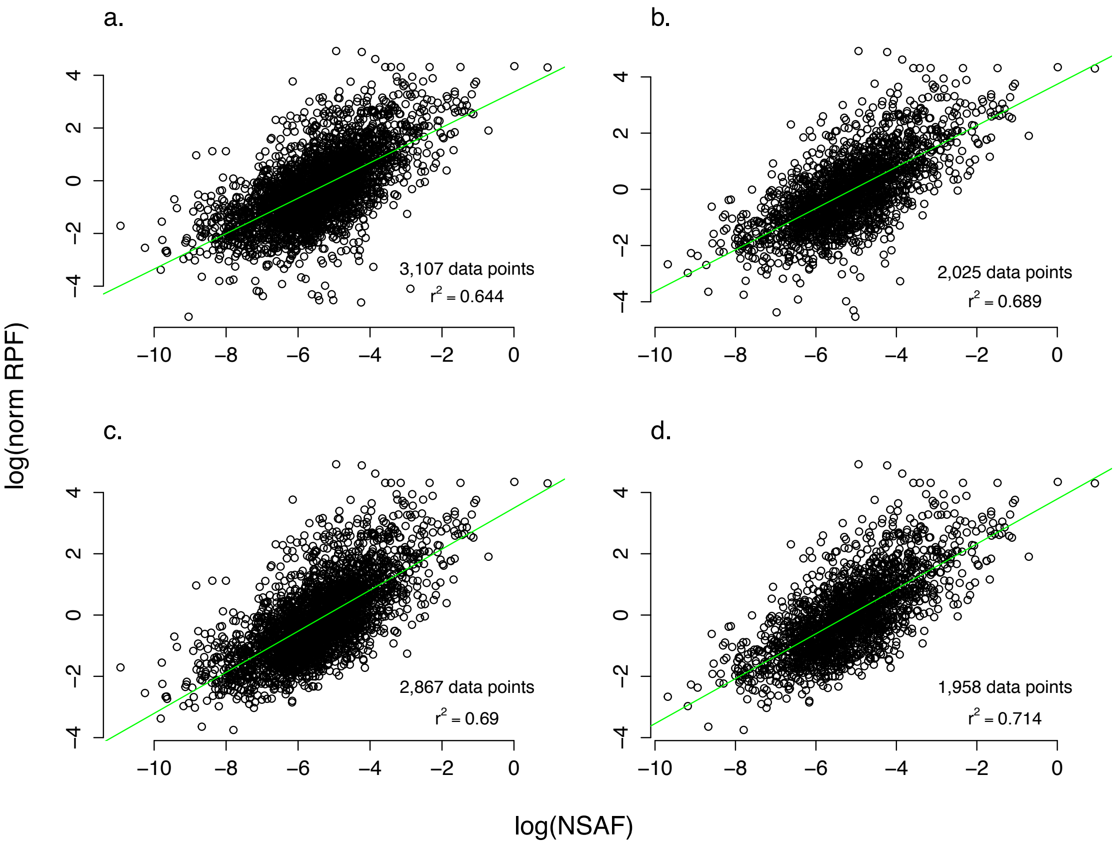
a.
3,107 data points
r2 = 0.644
b.
2,025 data points
r2 = 0.689
c.
2,867 data points
r2 = 0.69
d.
1,958 data points
r2 = 0.714
log(norm RPF)
log(NSAF)
−10
−8
−6
−4
−2
0
4
2
0
−2
−4

Human emPAI

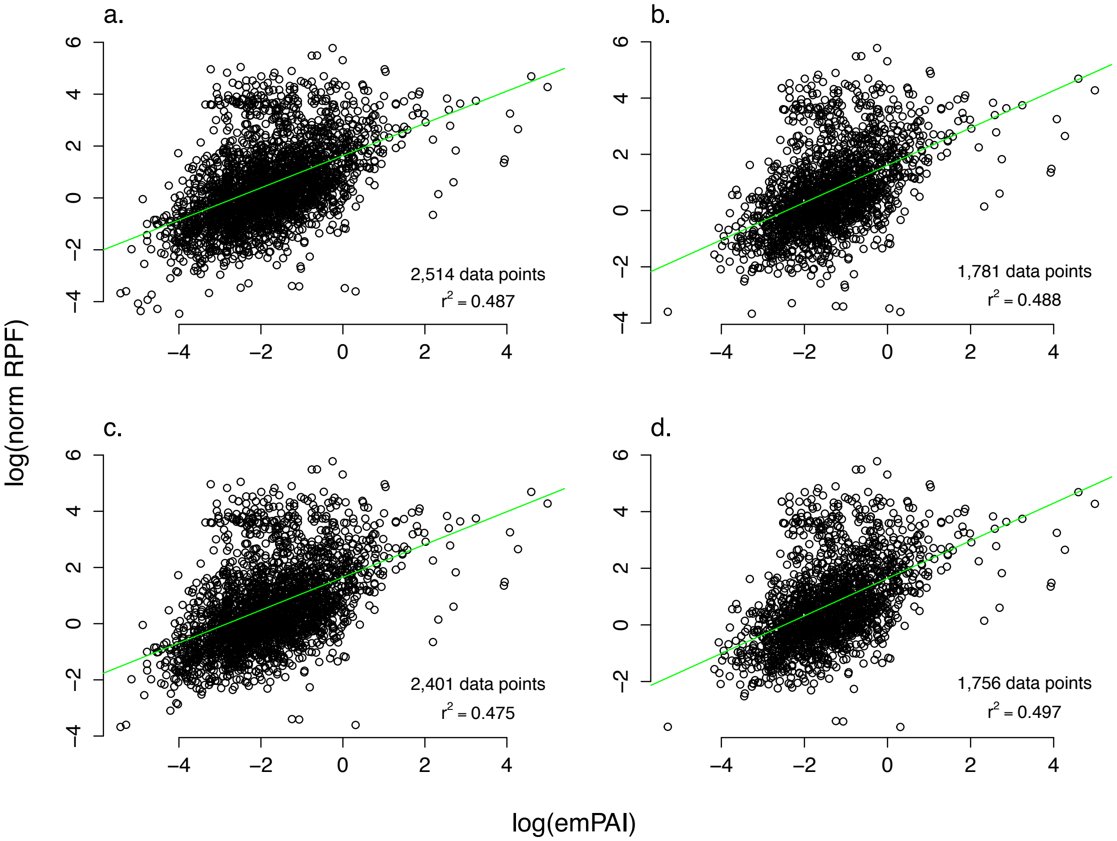
a.
b.
c.
d.
2,514 data points
$r^2 = 0.487$
1,781 data points
$r^2 = 0.488$
2,401 data points
$r^2 = 0.475$
1,756 data points
$r^2 = 0.497$
log(norm RPF)
log(emPAI)

Human NSAF

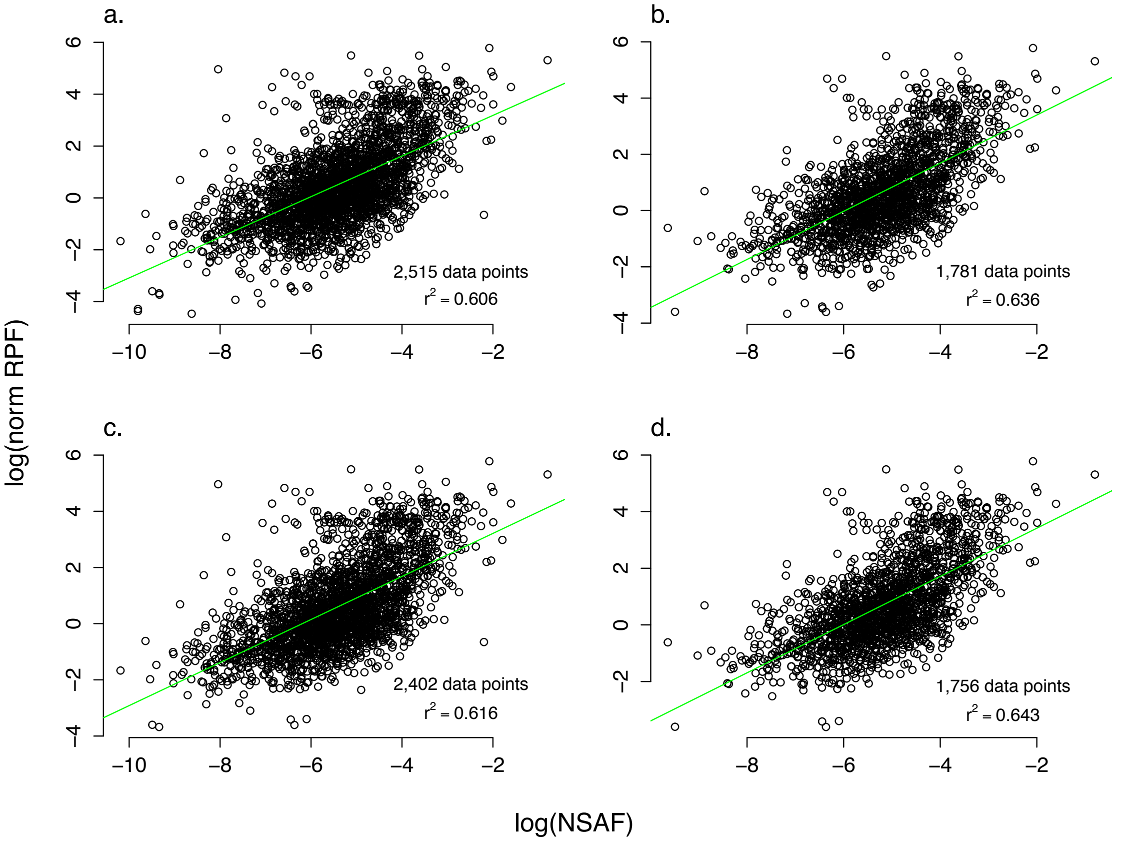

Supplementary Figure S5| Correlation plots of RPF counts (RIBO-seq) with protein abundance estimates based on emPAI and NSAF values for respectively human and mouse. **(a)** All annotated TIS (aTIS) transcripts. **(b)** Validated aTIS transcripts (i.e. transcripts with a spectral count ≥ 2). **(c)** aTIS transcripts with an RPF count ≥ 200. **(d)** Validated aTIS transcripts with an RPF count ≥ 200.

The regression line is shown in green. For each plot the number of data points used (i.e. the number of aTIS transcripts) as well as the corresponding Pearson correlation coefficient (r^2) is shown.

Supplementary Figure S6

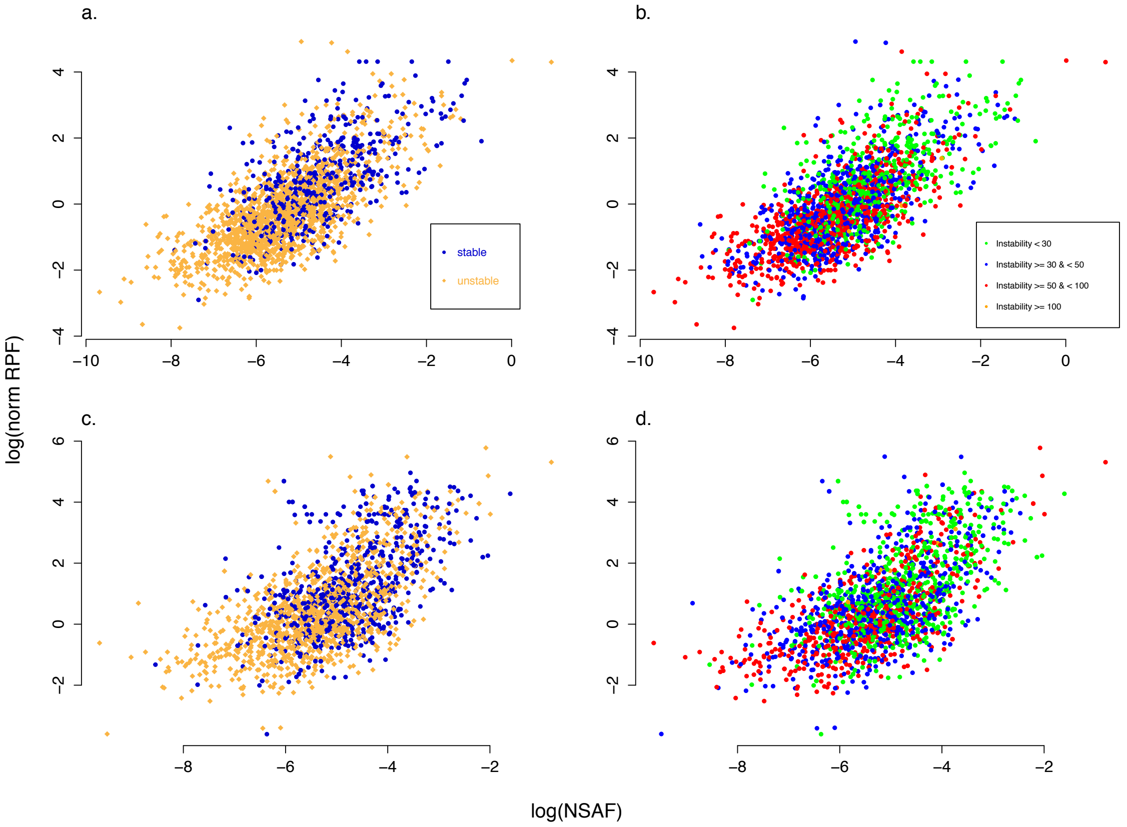

Supplementary Figure S6| Correlation plots of RPF-counts (RIBO-seq) with NSAF-based protein abundance estimates for validated (i.e. spectral count >= 2) aTIS transcripts with RPF count ≥ 200, with extra stability data annotation. **(a)** Mouse data is plotted; the instability indexes were determined with the ProtParam tool (http://web.expasy.org/protparam): proteins with an instability index < 40 were classified as stable and are shown in blue, whereas proteins with an instability index ≥ 40 were classified as unstable and are shown in orange. **(b)** Mouse data is plotted; proteins with an instability index < 30, ≥30 and < 50, ≥ 50 and < 100 or ≥ 100 are shown in green, blue, red and orange, respectively. Proteins with a high instability index are predicted to be more unstable. **(c)** Human data is plotted; similar to (a). **(d)** Human data is plotted; similar to (c).

Supplementary Figure S7

a. **HDGF_HUMAN**

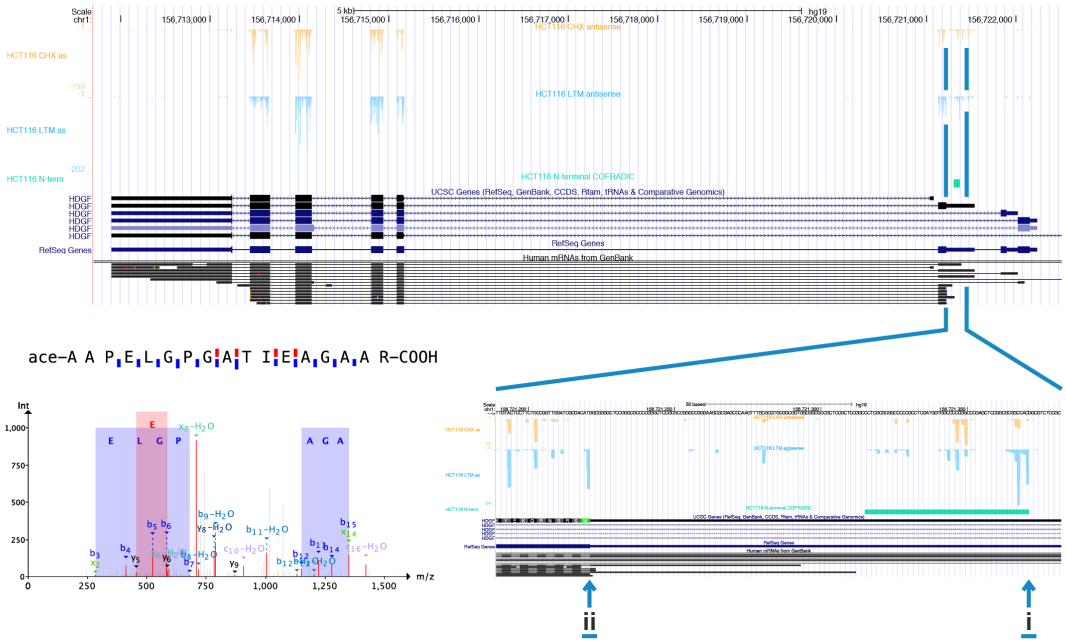

b. **HDGF_MOUSE**

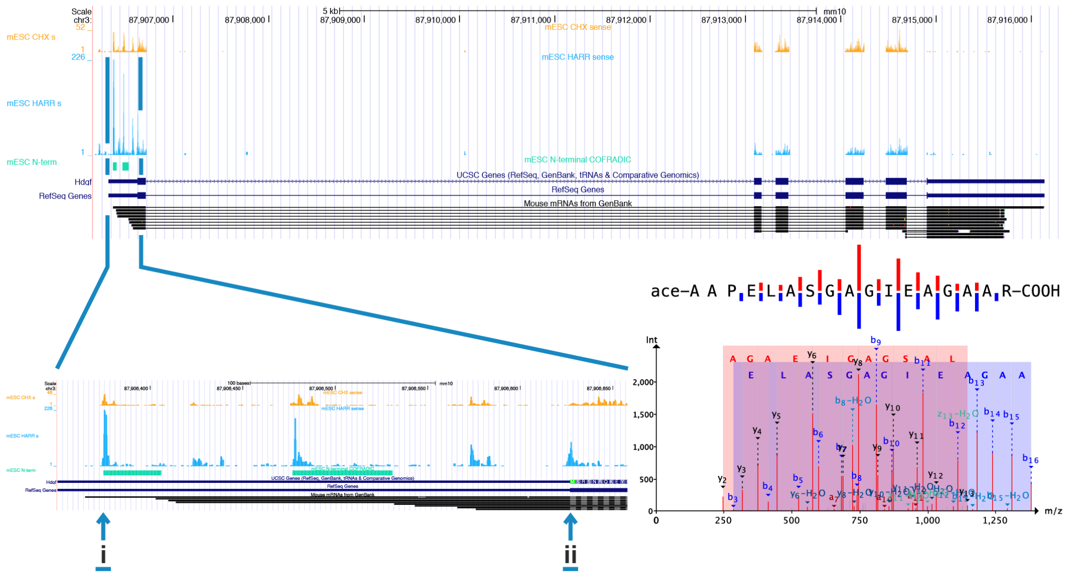

Supplementary Figure S7| Depiction of the HDGF 5'-extension predicted by RIBO-seq and identified using N-terminal COFRADIC for both the human (HDGF_HUMAN) and mouse (HDGF_MOUSE) orthologous proteoforms. The UCSC genome browser

was used to create the plots of the RIBO-seq and N-terminal COFRADIC data and the different browser tracks are from top to bottom: CHX treatment data, LTM/HARR treatment data, N-terminal COFRADIC data, UCSC genes, RefSeq genes and human/mouse mRNA from GenBank. The different start sites (i: alternative start site, ii: canonical start site) are clearly visible in the zoomed genome browser views. The MS/MS spectra and sequence fragmentations indicate the confidence and quality of the N-terminal peptide identifications. In both cases the N-terminus was found to be Nt-acetylated (ace-), a co-translational protein modification indicative of translation initiation, and the initiator methionine removed by the action of methionine aminopeptidase or MetAP.

Supplementary Figure S8

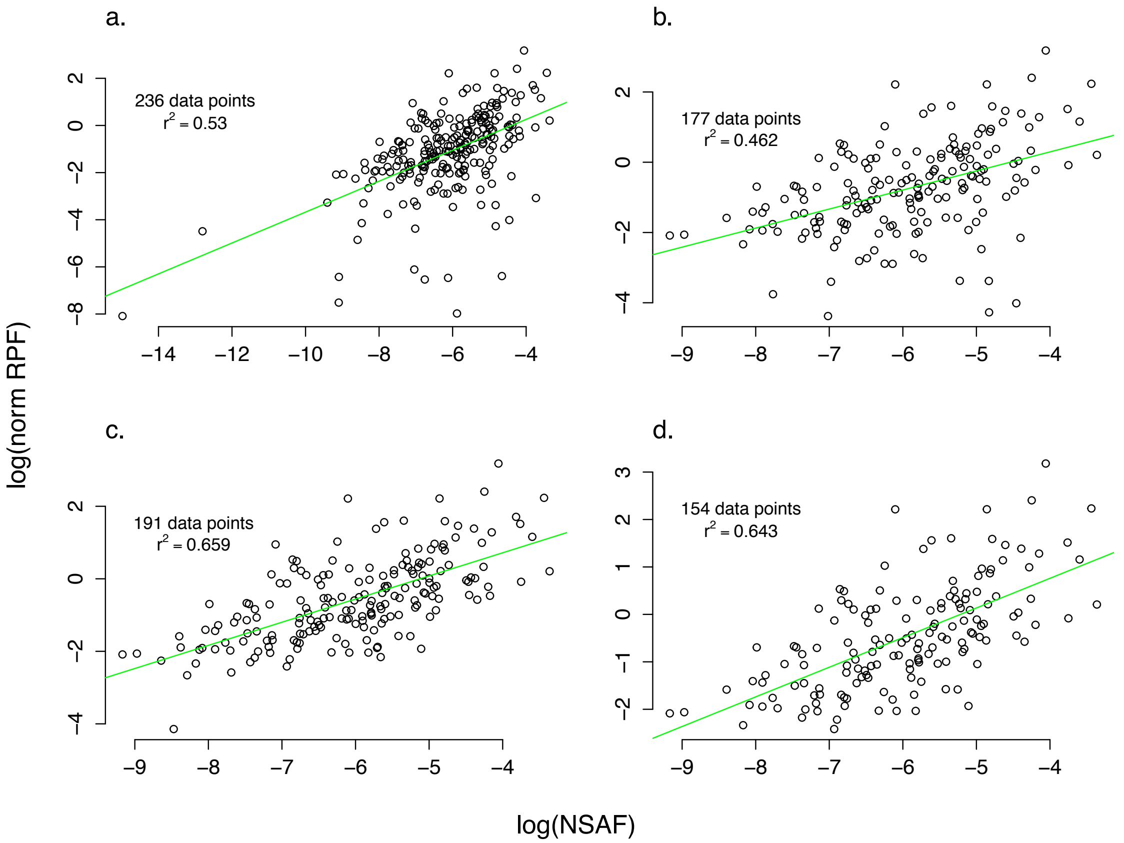

Supplementary Figure S8| Correlation plots of RPF-counts (RIBO-seq) with NSAF-based protein abundance estimates for the proteins uniquely identified in Swiss-Prot. These proteins were not derived from RIBO-seq data, because the LTM treatment and/or TIS calling failed to identify these TISs. Correlations could still be calculated as the CHX treatment did result in detectable coverage for these transcripts. **(a)** All annotated TIS (aTIS) transcripts. **(b)** Validated aTIS transcripts (i.e. transcripts with a spectral count ≥ 2). **(c)** aTIS transcripts with an RPF count ≥ 200. **(d)** Validated aTIS transcripts with an RPF count ≥ 200. The regression line is shown in green.

For each plot the number of data points used (i.e. the number of aTIS transcripts) as well as the corresponding Pearson correlation coefficient (r^2) is shown. The number of data points used in every plot is lower than the total number of unique Swiss-Prot identifications (253), because whenever a Swiss-Prot protein corresponded to multiple transcripts only the transcript with the highest normalized RPF value was used.

Supplementary Figure S9

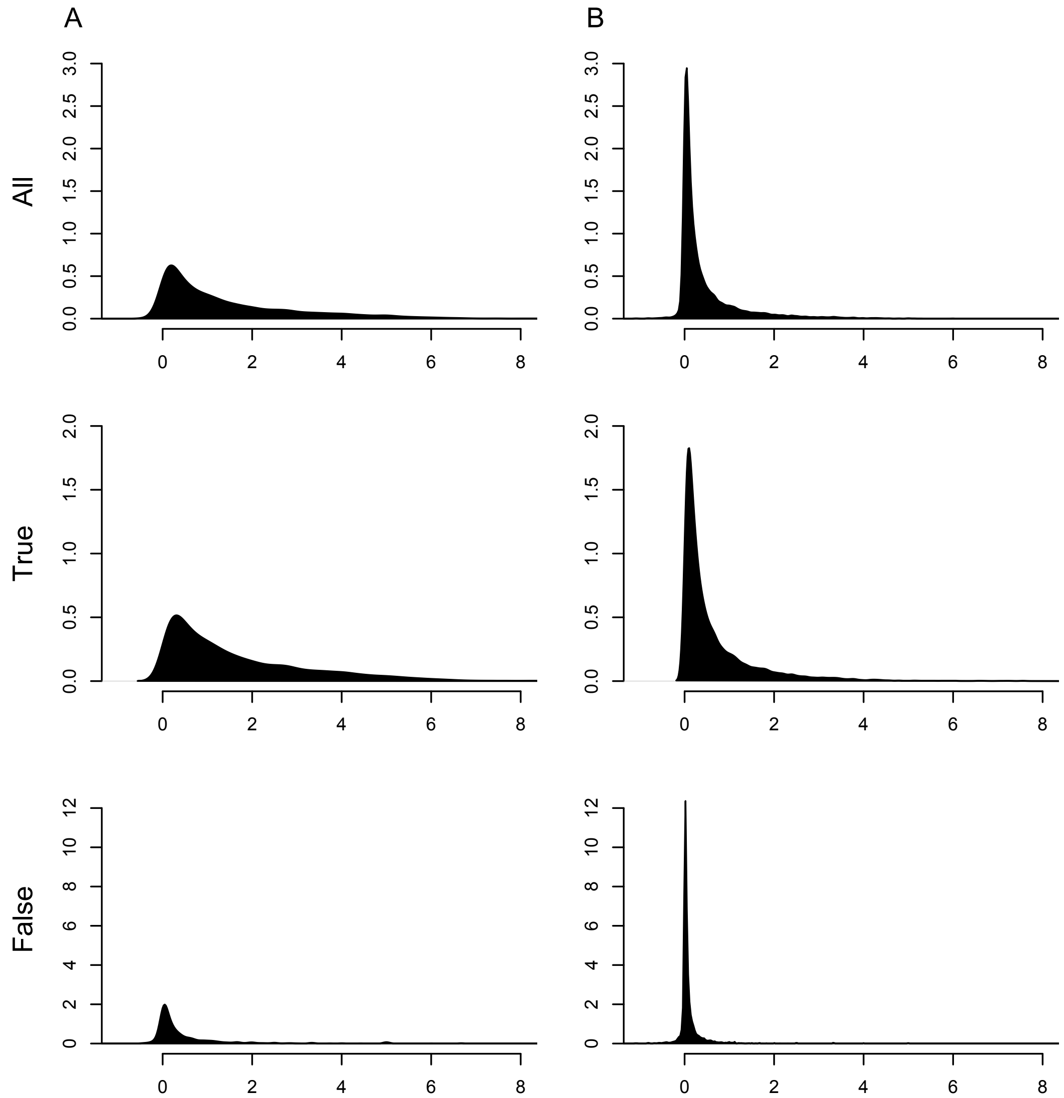

Supplementary Figure S9| $R_{LTM/HARR}$-R_{CHX} distribution for ribosome profile covered aTIS transcripts. These density plots show the distribution of the $R_{LTM/HARR}$-R_{CHX} parameter (see **Material and Methods** for a detailed description) for **(a)** the mouse (mESC) and **(b)** human (HCT116) aTIS transcripts. From top to bottom it represents the distribution of (i) all aTIS transcripts with ribosome profile coverage, (ii) all aTIS transcripts with ribosome profile coverage where the TIS is called by the rule-based algorithm (e.g. passing all TIS calling parameters) and (iii) all aTIS transcripts with ribosome profile coverage where the TIS is not called by the rule-based algorithm (e.g. not passing one or more TIS calling parameters). It is noticeable that the human $R_{LTM/HARR}$-R_{CHX} values are lower than those for the mouse data, possibly pointing to suboptimal LTM treatment and/or TIS calling or biases introduced in the library preparation of the sequencing experiment of the lactimidomycin treated HCT116 cell line sample.

Supplementary Figure S10

PROTEOFORMER workflow

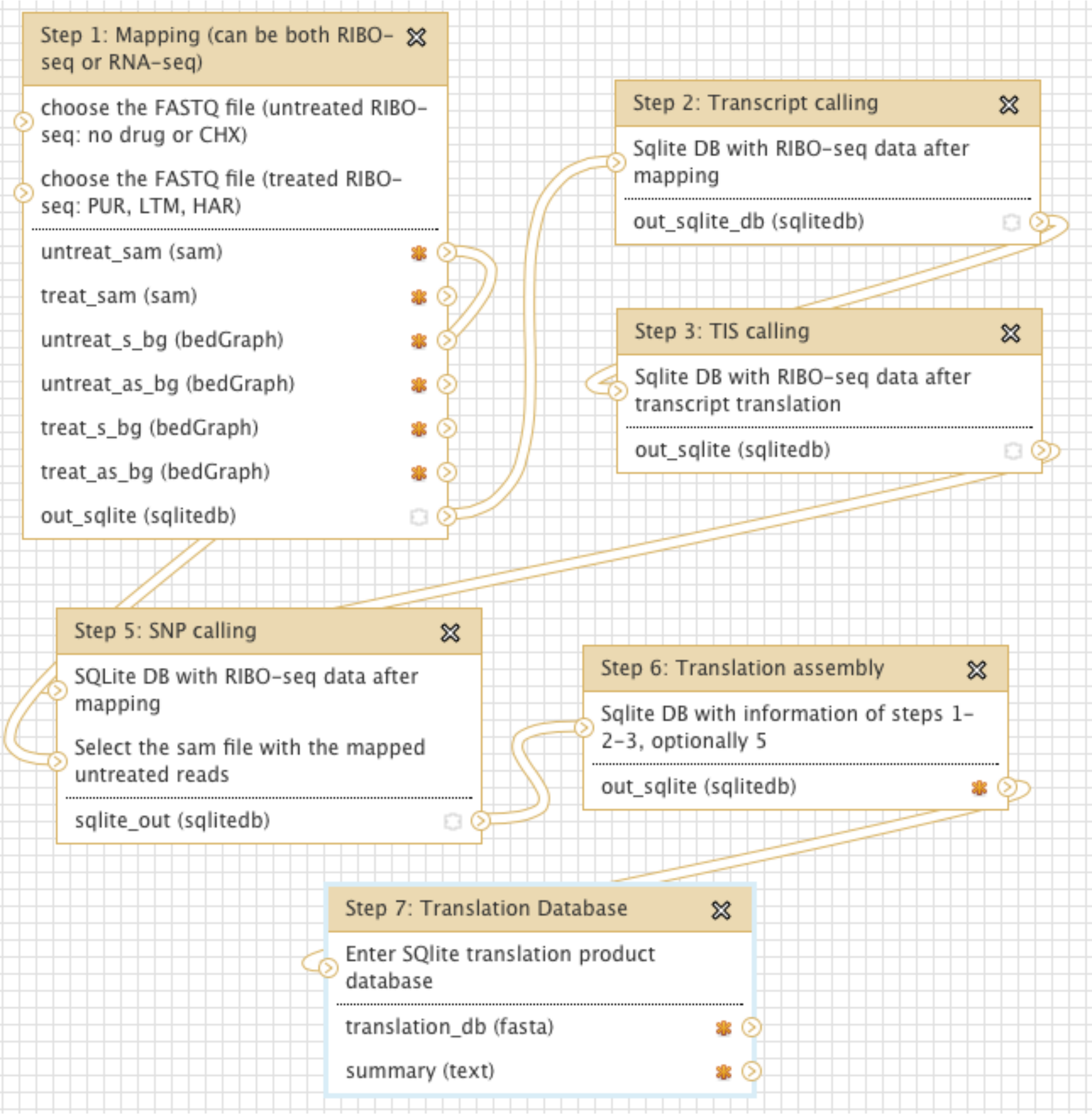

Quality Control workflow

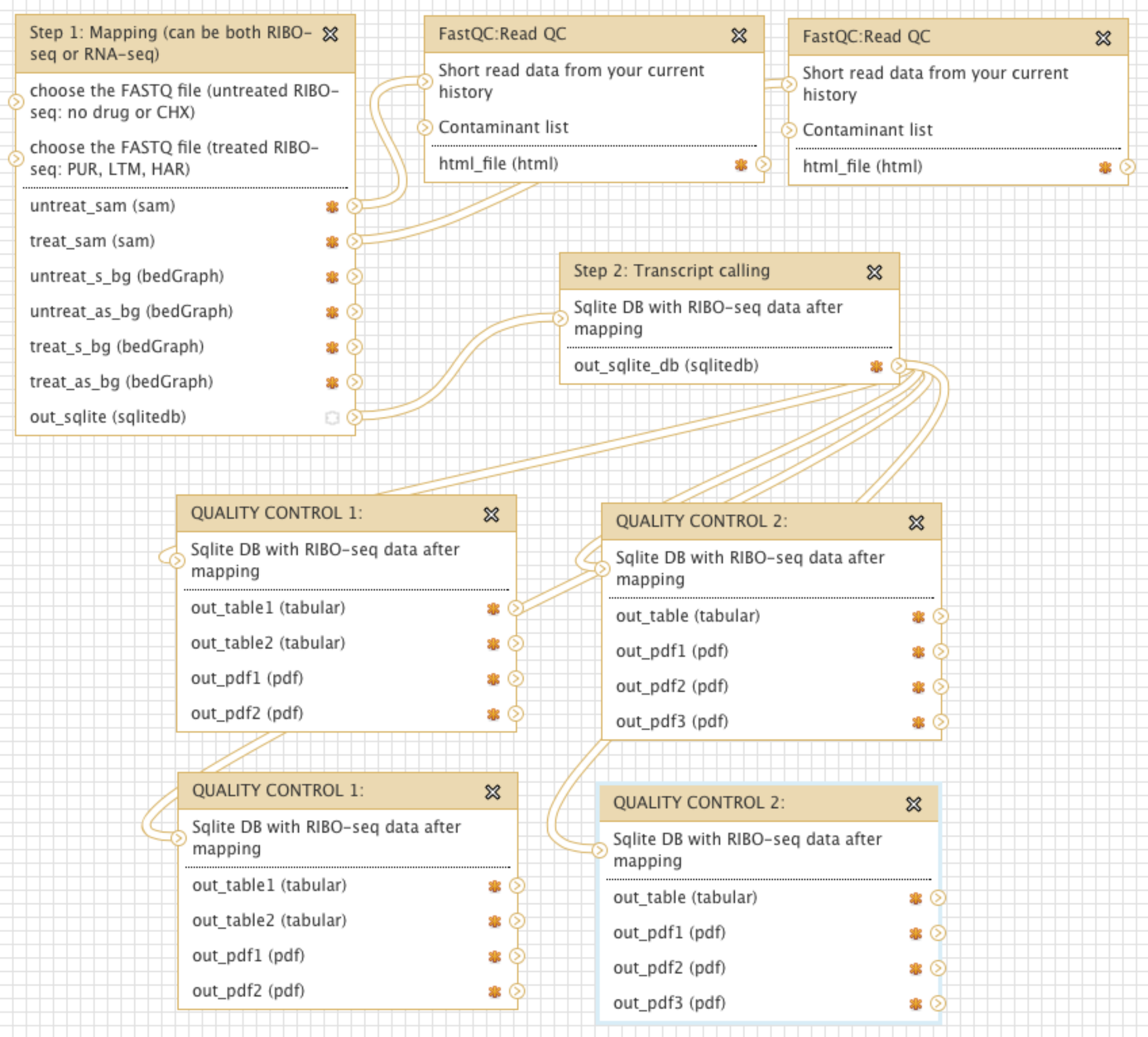

Supplementary Figure S10| (a) Screenshot depicting a Galaxy workflow containing all steps of the PROTEOFORMER tool pipeline in combination with the downstream MS/MS identification tools as depicted in **Fig. 1**. **(b)** Screenshot depicting a Galaxy workflow containing all steps of the PROTEOFORMER tool Quality Control in combination with FastQC Read Quality Control. The Galaxy workflows can also be downloaded from the PROTEOFORMER website (www.biobix.be/proteoformer).

Supplementary File S1

To further validate the uORF translation products, we inspected the peptide-to-spectrum matching (PSM) specifications, using the PeptideShaker tool (http://peptide-shaker.googlecode.com (7,14)). Afterwards we also investigated the corresponding gene model using the Ensembl genome browser (http://www.ensembl.org (15)) and applied the FGENESH gene structure prediction tool (http://www.softberry.com (16)) to scan the un-spliced genetic code (with 2000 bp upstream and downstream flanking sequence) for extra gene predictions. Clustal Omega (http://www.clustal.org/omega/ (17)) was used to align existing with newly identified proteoforms.

In total only a handful of uORF translation products were withheld:

	mESC shotgun	mESC Nterm	HCT116 shotgun	HCT116 Nterm
uORF proteoform	3	2	-	-

- Detected uORF translation products from mESC shotgun experiment:

ENSMUST00000145166_1_75436026_5UTR

PeptideShaker info:

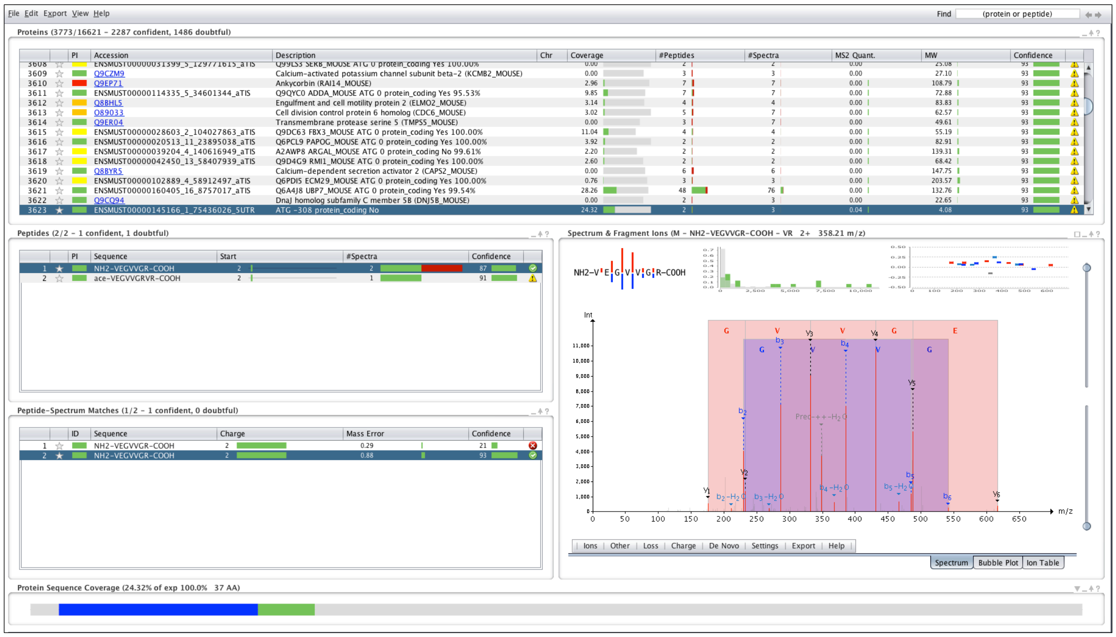

Ensembl info:

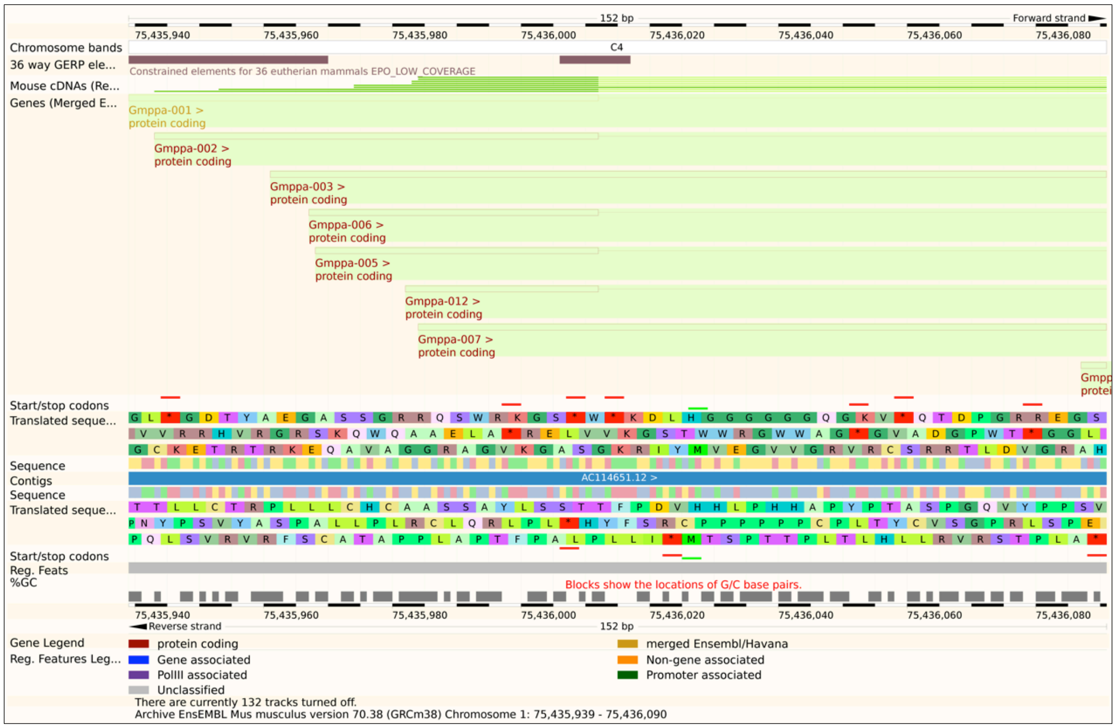

FGENESH info:

FGENESH 2.6 Prediction of potential genes in Mouse genomic DNA
Seq name: ENSMUSG00000033021|ENSMUST00000145166
Length of sequence: 6913
Number of predicted genes 1: in +chain 1, in -chain 0.
Number of predicted exons 6: in +chain 6, in -chain 0.
Positions of predicted genes and exons: Variant 1 from 1, Score:23.693184

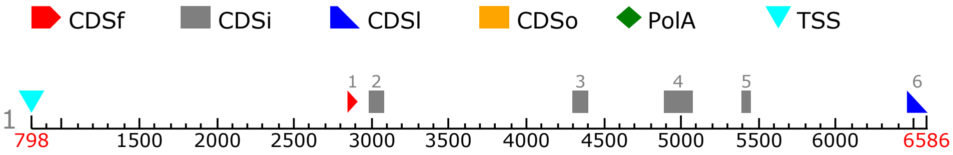


```
1 +      TSS        798                -3.19
1 +    1 CDSf      2839 -      2905    -0.07      2839 -      2904     66
1 +    2 CDSi      2977 -      3074     7.32      2979 -      3074     96
1 +    3 CDSi      4291 -      4394     6.80      4291 -      4392    102
1 +    4 CDSi      4882 -      5068     9.78      4883 -      5068    186
1 +    5 CDSi      5385 -      5444     3.67      5385 -      5444     60
1 +    6 CDSl      6452 -      6586    11.44      6452 -      6586    135
```

```
Predicted protein(s):
>FGENESH:[mRNA] 1 6 exon (s) 2839 - 6586 651 bp, chain +
ATGCTCAAAGCTGTGATTCTCATTGGAGGCCCCCAGAAGGGTGAGGAGATGGGGGACAGG
GGAGCGGGGACTCGCTTCAGGCCTTTGTCTTTTGAGGTGCCCAAACCTCTGTTTCCTGTG
GCAGGCGTTCCCATGATCCAGCACCATATAGAAGCCTGTGCCCAGGTCCCAGGGATGCAG
GAGATTCTTCTCATTGGCTTCTACCAGCCTGATGAGGCCCTCACCCAGTTCCTGGAAGCT
GCCCAGCAGGAGTTTAACCTTCCAGTCAGGTACCTGCAGGAGTTTGCCCCCCTCGGCACA
GGGGGTGGCCTCTACCATTTTCGGGACCAGATCCTGGCTGGGGCACCTGAGGCCTTCTTC
GTGCTCAATGCTGACGTCTGCTCTGACTTCCCCTTGAGCGCCATGTTGGAGGCTCACAGG
CGCCAGCGCCACCCTTTCTTACTCCTTGGCACCACGGCTAACAGGACACAATCCCTCAAC
TACGGCTGCATCGTTGAGAATCCACAGACTCATGAGGTTCTGCACTATGTGGAGAAACCC
AGCACCTTTATCAGTGACATCATCAACTGTGGCATCTACCTTTTCTCCCCAGAAGCCCTG
AAGCCTCTCCGGGATGTTTTCCAGCGTAACCAACAGGATGGGCAACTGTGA
>FGENESH: 1 6 exon (s) 2839 - 6586 216 aa, chain +
MLKAVILIGGPQKGEEMGDRGAGTRFRPLSFEVPKPLFPVAGVPMIQHHIEACAQVPGMQ
EILLIGFYQPDEALTQFLEAAQQEFNLPVRYLQEFAPLGTGGGLYHFRDQILAGAPEAFF
VLNADVCSDFPLSAMLEAHRRQRHPFLLLGTTANRTQSLNYGCIVENPQTHEVLHYVEKP
STFISDIINCGIYLFSPEALKPLRDVFQRNQQDGQL
```

The fact that Fgenesh wasn't able to predict an extra gene model including the 5' uORF and that the spectral matching information is good, this could point to a uORF identification.

ENSMUST00000034720_9_71485905_5UTR

PeptideShaker info:

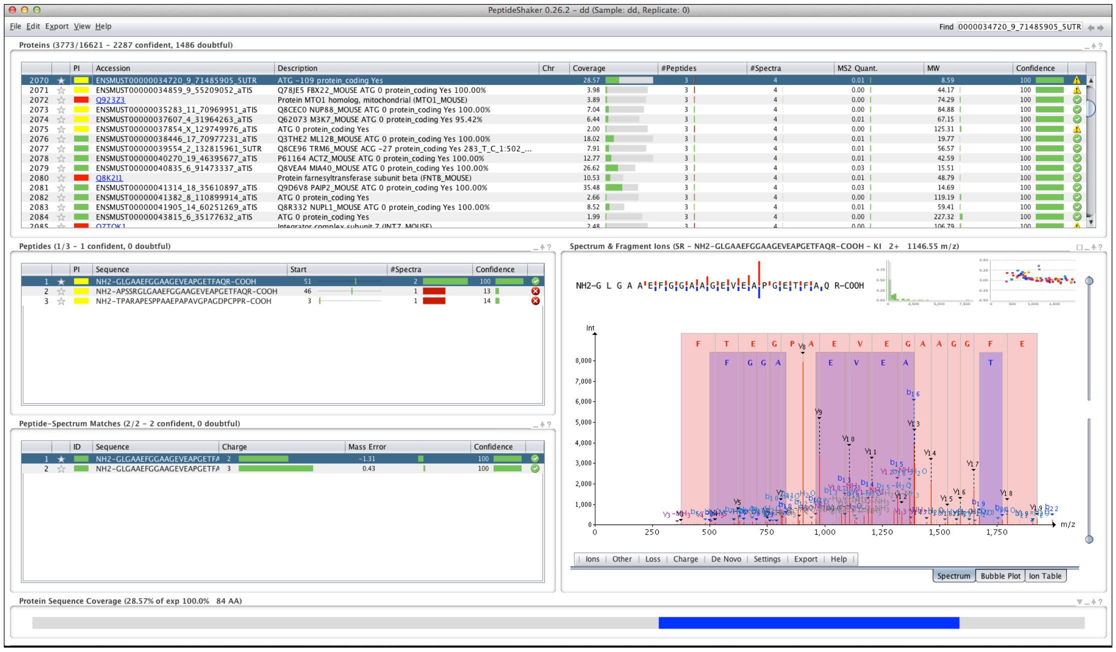

Ensembl info:

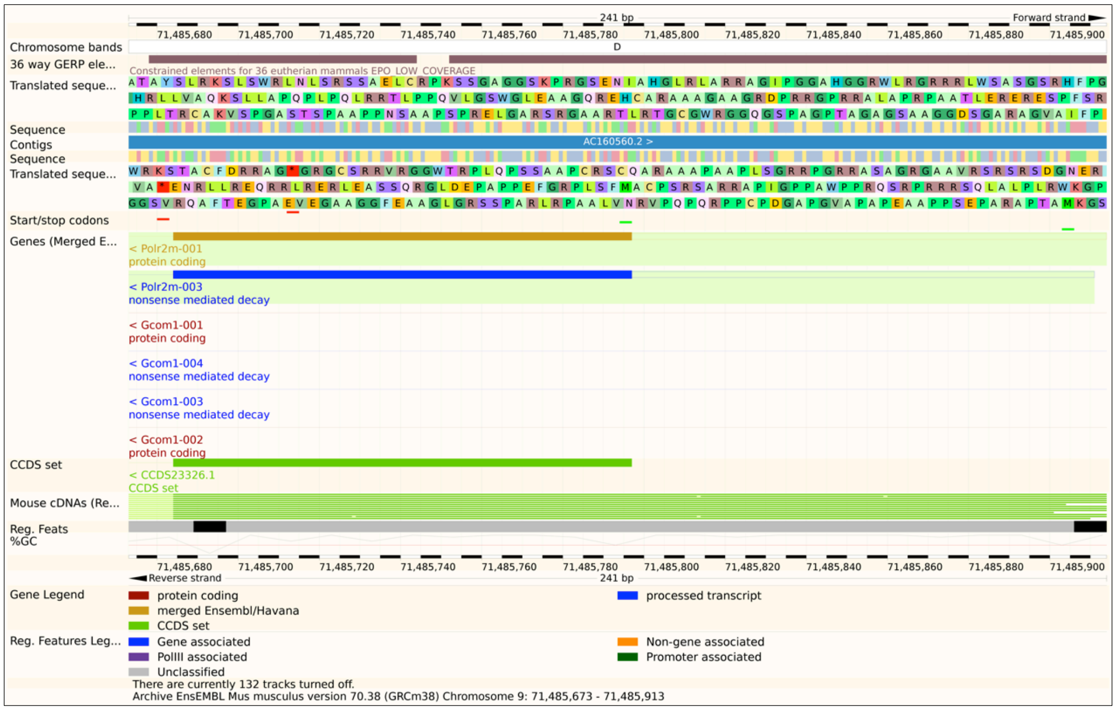

FGENESH info:

FGENESH 2.6 Prediction of potential genes in Mouse genomic DNA
Seq name:
ENSMUSG00000032199|ENSMUST00000163972;ENSMUST00000034720;ENSMUST0000[illegible]
Length of sequence: 11499
Number of predicted genes 1: in +chain 1, in -chain 0.
Number of predicted exons 5: in +chain 5, in -chain 0.
Positions of predicted genes and exons: Variant 1 from 1, Score:50.943002

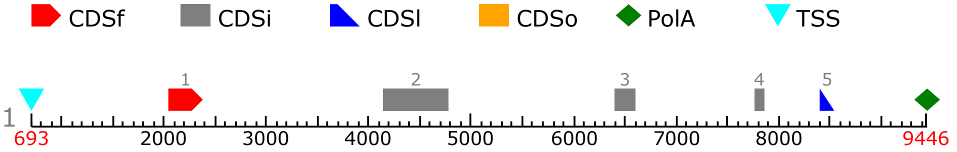

1 +		TSS	693			-5.39			
1 +	1	CDSf	2031 -	2368	21.11	2031 -	2366	336
1 +	2	CDSi	4131 -	4769	25.00	4132 -	4767	636
1 +	3	CDSi	6390 -	6594	8.75	6391 -	6594	204
1 +	4	CDSi	7759 -	7854	-0.00	7759 -	7854	96
1 +	5	CDSl	8396 -	8539	6.80	8396 -	8539	144
1 +		PolA	9446		0.93			

```
Predicted protein(s):
>FGENESH:[mRNA] 1 5 exon (s) 2031 - 8539 1422 bp, chain +
ATGGCGACTCCCGCTCGCGCTCCAGAGTCGCCGCCGGCCGCGGAGCCAGCGCCCGCCGTG
GGCCCCGCCGGGGATCCCTGCCCGCCGCGCCAGCCGCAGCCCGTGCGCAATGTTCTCGCT
GCCCCGCGGCTTCGAGCCCCCAGCTCCCGAGGACTTGGGGCGGCAGAGTTCGGCGGAGCT
GCGGGAGAGGTTGAGGCGCCAGGAGAGACTTTTGCGCAACGAGTAAGCGGTGGCCCTGGG
GTCGCGGTTGCGGGGGATCGGGATCAGGGAACAGGCTCTCAGGGATCGGGATCCGGGGAC
TTGGTCTCCCGCCCCGCCCCGCGGCCTGGCGCGCGAAGAAAATTCATTTGCAAATTGCCC
GACAAAGGTAAAAAGATCTCAGACACAGTTGCCAAACTGAAAGCTGCCATTTCAGAACGT
GAAGAGGTTAGAGGGAGAAGTGAACTGTTTCATCCTGTTAGTGTAGACTGTAAGCTAAGG
CAAAAAGCAACCACAAGAGCTGACACCGATGTAGACAAGGCCCAGAGTTCTGACCTGATG
CTTGATACTTCATCATTAGATCCTGACTGTTCCTCAATAGACATTAAGTCATCTAAATCA
ACCTCAGAAACACAGGGACCTACACATCTCACTCACAGAGGCAATGAAGAGACTTTGGAG
GCTGGCTACACAGTAAACAGCAGCCCAGCTGCCCACATCCGAGCCCGGGCGCCCTCATCC
GAAGTTAAGGAGCATCTCCCCCAGCACTCTGTTTCAAGTCAAGAGGAAGAGATCTCCAGC
AGCATCGACAGTCTCTTCATCACTAAATTGCAAAAAATCACAATTGCAGACCAGAGTGAA
CCCTCAGAAGAAAACACCAGCACTGAGAACTTTCCAGAACTGCAGAGTGAGACTCCTAAG
AAGCCTCATTACATGAAAGTGCTAGAAATGCGAGCCAGAAACCCAGTGCCCCCTCCTCAT
AAGTTTAAGACCAATGTGTTACCCACACAACAGAGTGACTCACCAAGTCATTGTCAGAGG
GGCCAGTCTCCTGCTTCCTCAGAAGAGCAGCGACGAAGGGCTAGGCAGCATCTTGATGAT
ATCACAGCAGCGCGCCTCCTTCCGCTCCACCACCTGCCTGCACAGCTGCTTTCCATAGAA
GAGTCGCTGGCCCTGCAGAGGGAGCAGAAGCAGAATTATGAGAATAGTAATTATGATACC
AATTATGCCTACCCATATATCGTGGGCAGAGAGGAAGGACCTGCTATGGGCGGTACAGAA
GTGTGGGTGGTACAGAAGGAGATGCAGGCAAAGCTCGCAGCACAGAAACTGGCCGAGAGA
CTGAATATTAAAATGCAGAGCTACAATCCAGAAGGGGAGTCTTCAGGGAGATACCGAGAA
GTGAGGGACGAAGCTGATGCCCAGTCCTCGGATGAGTGCTGA
>FGENESH: 1 5 exon (s) 2031 - 8539 473 aa, chain +
MATPARAPESPPAAEPAPAVGPAGDPCPPRQPQPVRNVLAAPRLRAPSSRGLGAAEFGGA
AGEVEAPGETFAQRVSGGPGVAVAGDRDQGTGSQGSGSGDLVSRPAPRPGARRKFICKLP
DKGKKISDTVAKLKAAISEREEVRGRSELFHPVSVDCKLRQKATTRADTDVDKAQSSDLM
LDTSSLDPDCSSIDIKSSKSTSETQGPTHLTHRGNEETLEAGYTVNSSPAAHIRARAPSS
EVKEHLPQHSVSSQEEEISSSIDSLFITKLQKITIADQSEPSEENTSTENFPELQSETPK
KPHYMKVLEMRARNPVPPPHKFKTNVLPTQQSDSPSHCQRGQSPASSEEQRRRARQHLDD
ITAARLLPLHHLPAQLLSIEESLALQREQKQNYENSNYDTNYAYPYIVGREEGPAMGGTE
VWVVQKEMQAKLAAQKLAERLNIKMQSYNPEGESSGRYREVRDEADAQSSDEC
```

Clustal Omega alignment:

```
FGENESH:                 MATPARAPESPPAAEPAPAVGPAGDPCPPRQPQPVRNVLAAPRLRAPSSRGLGAAEFGGA
ENSMUST00000163972       ------------------------------------------------------------
ENSMUST00000034720       ------------------------------------------------------------

FGENESH:                 AGEVEAPGETFAQRVSGGPGVAVAGDRDQGTGSQGSGSGDLVSRPAPRPGARRKFICKLP
ENSMUST00000163972       ------------------------------------------------------------
ENSMUST00000034720       ---------MFS----LPRGFEPPAPEDL--GRQSSAELRERLRRQERLLRNEKFICKLP

FGENESH:                 DKGKKISDTVAKLKAAISEREEVRGRSELFHPVSVDCKLRQKATTRADTDVDKAQSSDLM
ENSMUST00000163972       -----------------------------------------------------------M
ENSMUST00000034720       DKGKKISDTVAKLKAAISEREEVRGRSELFHPVSVDCKLRQKATTRADTDVDKAQSSDLM
                                                                                    *
FGENESH:                 LDTSSLDPDCSSIDIKSSKSTSETQGPTHLTHRGNEETLEAGYTVNSSPAAHIRARAPSS
ENSMUST00000163972       LDTSSLDPDCSSIDIKSSKSTSETQGPTHLTHRGNEETLEAGYTVNSSPAAHIRARAPSS
ENSMUST00000034720       LDTSSLDPDCSSIDIKSSKSTSETQGPTHLTHRGNEETLEAGYTVNSSPAAHIRARAPSS
                         ************************************************************

FGENESH:                 EVKEHLPQHSVSSQEEEISSSIDSLFITKLQKITIADQSEPSEENTSTENFPELQSETPK
ENSMUST00000163972       EVKEHLPQHSVSSQEEEISSSIDSLFITKLQKITIADQSEPSEENTSTENFPELQSETPK
ENSMUST00000034720       EVKEHLPQHSVSSQEEEISSSIDSLFITKLQKITIADQSEPSEENTSTENFPELQSETPK
                         ************************************************************
FGENESH:                 KPHYMKVLEMRARNPVPPPHKFKTNVLPTQQSDSPSHCQRGQSPASSEEQRRRARQHLDD
ENSMUST00000163972       KPHYMKVLEMRARNPVPPPHKFKTNVLPTQQSDSPSHCQRGQSPASSEEQRRRARQHLDD
ENSMUST00000034720       KPHYMKVLEMRARNPVPPPHKFKTNVLPTQQSDSPSHCQRGQSPASSEEQRRRARQHLDD
                         ************************************************************
FGENESH:                 ITAARLLPLHHLPAQLLSIEESLALQREQKQNYENSNYDTNYAYPYIVGREEGPAMGGTE
ENSMUST00000163972       ITAARLLPLHHLPAQLLSIEESLALQREQKQNYE--------------------------
ENSMUST00000034720       ITAARLLPLHHLPAQLLSIEESLALQREQKQNYE--------------------------
                         **********************************

FGENESH:                 VWVVQKEMQAKLAAQKLAERLNIKMQSYNPEGESSGRYREVRDEADAQSSDEC
ENSMUST00000163972       ------EMQAKLAAQKLAERLNIKMQSYNPEGESSGRYREVRDEADAQSSDEC
ENSMUST00000034720       ------EMQAKLAAQKLAERLNIKMQSYNPEGESSGRYREVRDEADAQSSDEC
                               ***********************************************
```

Fgenesh was able to predict a 5’ extended gene product including the uORF sequence, so this could also point to a 5’ extended proteoform.

ENSMUST00000109554_15_81745101_5UTR

PeptideShaker info:

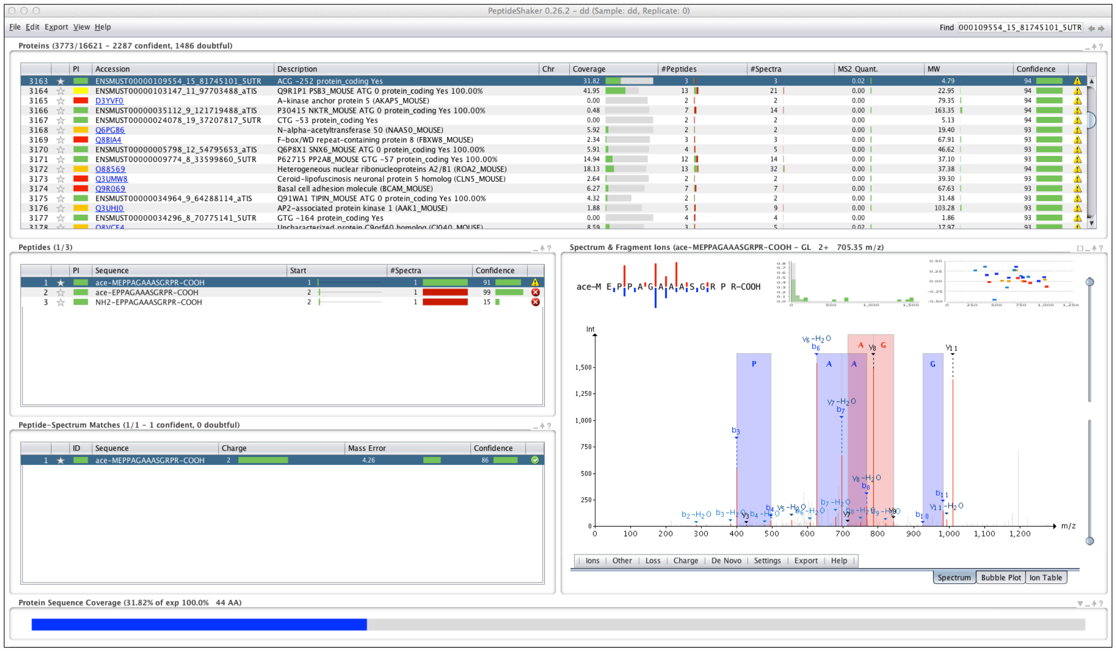

Ensembl info:

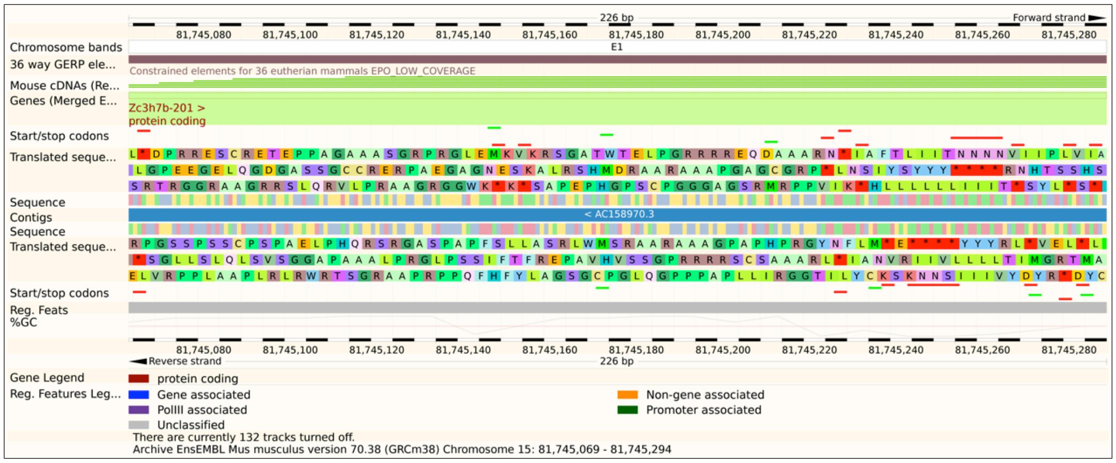

Due to the rather high peptide mass error (-4.25Da) and uncommon near-cognate start site (Threonine), this identification is doubtful.

ENSMUST00000132969_11_59449969_5UTR

PeptideShaker info:

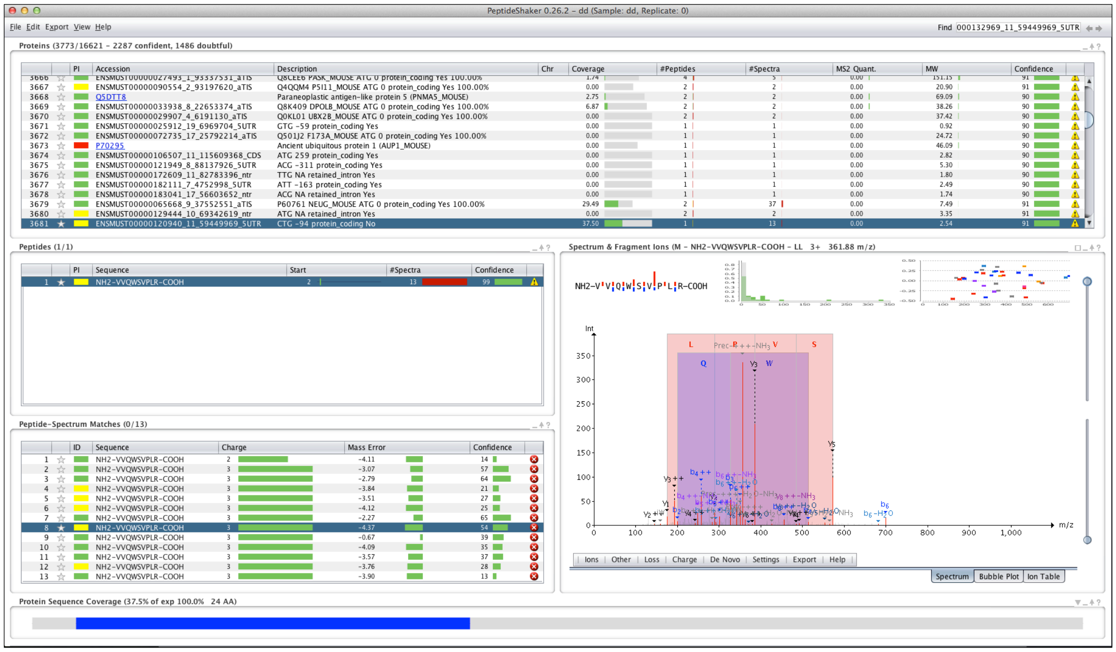

Since confident PSM was obtained, this identification was not retained.

- Detected uORF translation products from mESC Nterm experiment:

ENSMUST00000050476_18_36679609_5UTR

PeptideShaker info:

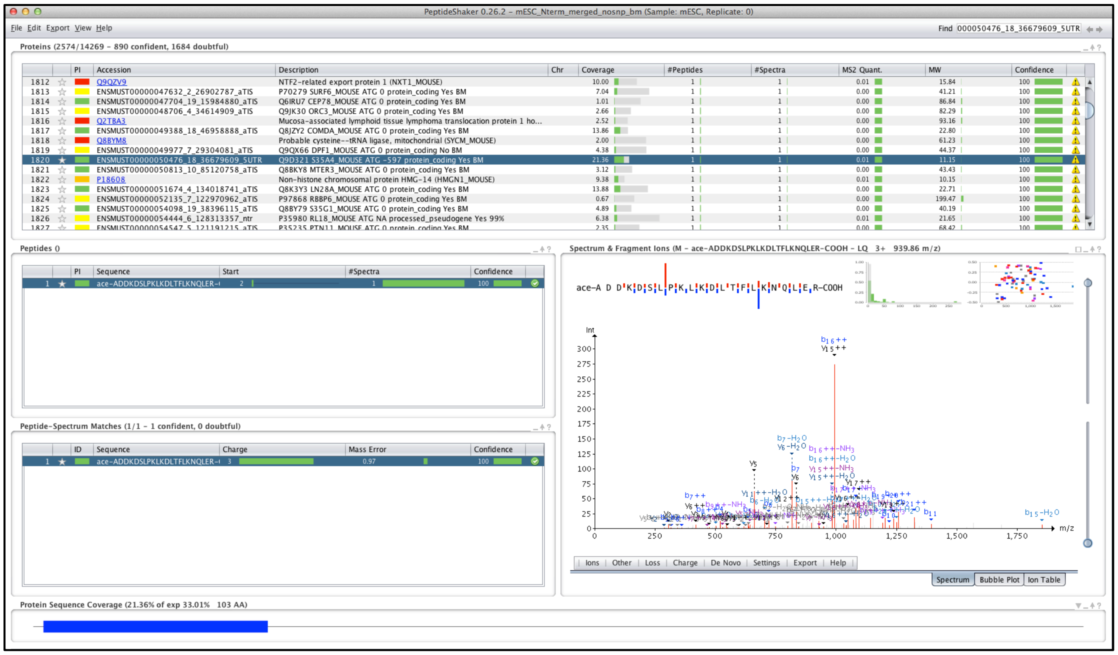

Ensembl info:

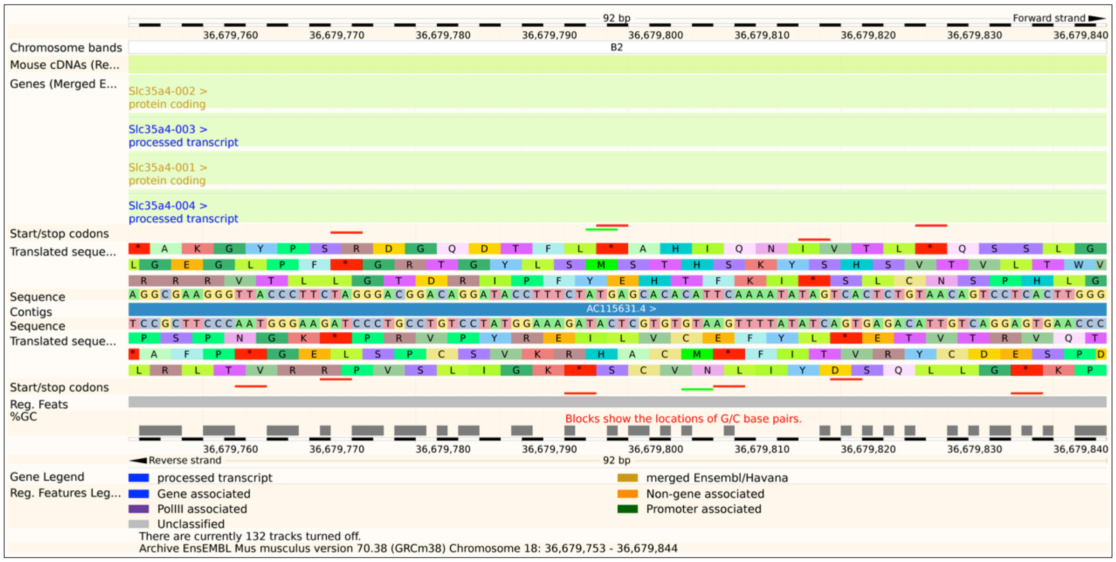

FGENESH info:

FGENESH 2.6 Prediction of potential genes in Mouse genomic DNA
Seq name:
ENSMUSG00000033272|ENSMUST00000050476;ENSMUST00000170288;ENSMUST0000
Length of sequence: 8220
Number of predicted genes 2: in +chain 2, in -chain 0.
Number of predicted exons 4: in +chain 4, in -chain 0.
Positions of predicted genes and exons: Variant 1 from 1, Score:81.279681

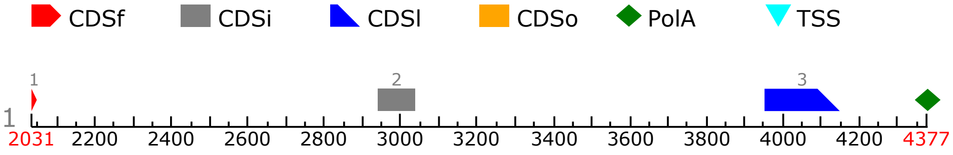

1	+	1	CDSf	2031	-	2045	12.14	2031 -	2045	15
1	+	2	CDSi	2938	-	3036	15.05	2938 -	3036	99
1	+	3	CDSl	3950	-	4147	15.78	3950 -	4147	198
1	+		PolA	4377			-6.88			

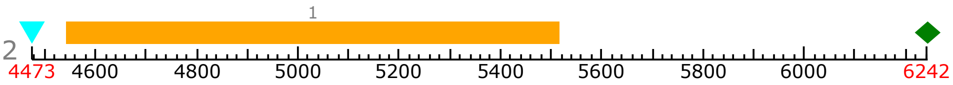

2	+		TSS	4473			-12.19			
2	+	1	CDSo	4541	-	5515	69.32	4541 -	5515	975
2	+		PolA	6242			0.93			

Predicted protein(s):
>FGENESH:[mRNA] 1 3 exon (s) 2031 - 4147 312 bp, chain +
ATGGCGGATGACAAGGATTCTCTGCCCAAGCTTAAGGACCTGACATTTCTCAAGAACCAG
CTGGAGCGCCTACAGCAGCGTGTGGAAGGTGAAGTCAACAGTGGCGTAGGCCAGGATGGC
TCCCTCTTGTCCTCCCCATTCTTCAAGGGCTTCCTGGCAGGATACGTGGTGGCCAAACTG
AGGGCATCAGCAGTATTGGGCTTTGCGGTGGGCACTTGCACTGGCATCTATGCAGCTCAG
GCATATGCCGTACCCAACGTGGAGAAGGCACTGAAGAACTACTTTAGGTCACTACGGAAG
GGGCCTGACTAG
>FGENESH: 1 3 exon (s) 2031 - 4147 103 aa, chain +
MADDKDSLPKLKDLTFLKNQLERLQQRVEGEVNSGVGQDGSLLSSPFFKGFLAGYVVAKL
RASAVLGFAVGTCTGIYAAQAYAVPNVEKALKNYFRSLRKGPD
>FGENESH:[mRNA] 2 1 exon (s) 4541 - 5515 975 bp, chain +
ATGAGTGTAGAAGATGGGGGCGTGCCAGGCCTAGCCCGCCCAAGACAGGCTCGCTGGACC
CTGTTGCTCTTCCTGTCCACTGCCATGTATGGTGCCCATGCACCGTTCTTAGCACTGTGC
CATGTGGATGGCCGAGTGCCCTTCCGGCCCTCCTCAGCTGTGTTACTCACTGAGCTGACC
AAGCTCCTGTTGTGCGCCTTCTCCCTCCTGGTAGGCTGGCAAACATGGCCCCAGGGCACG
CCACCCTGGCGCCAGGCTGTGCCTTTTGCACTGTCAGCCCTGCTCTATGGCGCCAACAAC
AACCTGGTGATTTATCTGCAGCGCTACATGGACCCCAGCACCTATCAGGTGCTGAGCAAT
CTCAAGATTGGAAGCACAGCTCTATTGTACTGCCTCTGCCTTGGGCATCGTCTCTCTGCG
CGTCAGGGCTTGGCGCTGCTGCTGCTGATGGCTGCAGGAGCCTGCTATGCATCAGGTGGC
TTTCAGGAACCTGTGAACACCCTTCCTGGGCCCGCGTCAGCAGCTGGAGCCCATCCCATG

```
CCCTTGCATATCACTCCACTGGGACTTCTGCTCCTCATCCTATACTGCCTCATCTCCGGC
TTGTCCTCCGTGTACACAGAGCTGATCATGAAGCGACAGCGGTTGCCCTTGGCTCTTCAG
AACCTCTTCCTCTACACTTTTGGGGTGATCCTGAACTTTGGACTGTATGCTGGCAGTGGC
CCAGGCCCGGGCTTCCTGGAGGGCTTCTCTGGATGGGCAGTGCTTGTGGTGCTGAACCAA
GCAGTCAATGGGCTGCTCATGTCGGCTGTCATGAAGCATGGCAGCAGCATCACACGCCTC
TTCATCGTGTCCTGCTCGCTCGTGGTCAACGCTGTGCTGTCGGCGGTGCTGCTCCAGCTG
CAGCTCACGGCCATCTTCTTCCTGGCCGCACTGCTCATCGGTCTGGCTGTGTGCTTGTAC
TATGGTAGCCCCTAA
>FGENESH: 2 1 exon (s) 4541 - 5515 324 aa, chain +
MSVEDGGVPGLARPRQARWTLLLFLSTAMYGAHAPFLALCHVDGRVPFRPSSAVLLTELT
KLLLCAFSLLVGWQTWPQGTPPWRQAVPFALSALLYGANNNLVIYLQRYMDPSTYQVLSN
LKIGSTALLYCLCLGHRLSARQGLALLLLMAAGACYASGGFQEPVNTLPGPASAAGAHPM
PLHITPLGLLLLILYCLISGLSSVYTELIMKRQRLPLALQNLFLYTFGVILNFGLYAGSG
PGPGFLEGFSGWAVLVVLNQAVNGLLMSAVMKHGSSITRLFIVSCSLVVNAVLSAVLLQL
QLTAIFFLAALLIGLAVCLYYGSP
```

The spectral matching is good and furthermore Fgenesh was able to predict an extra 5’ uORF coding sequence.

ENSMUST00000027264_1_53352619_5UTR

PeptideShaker info:

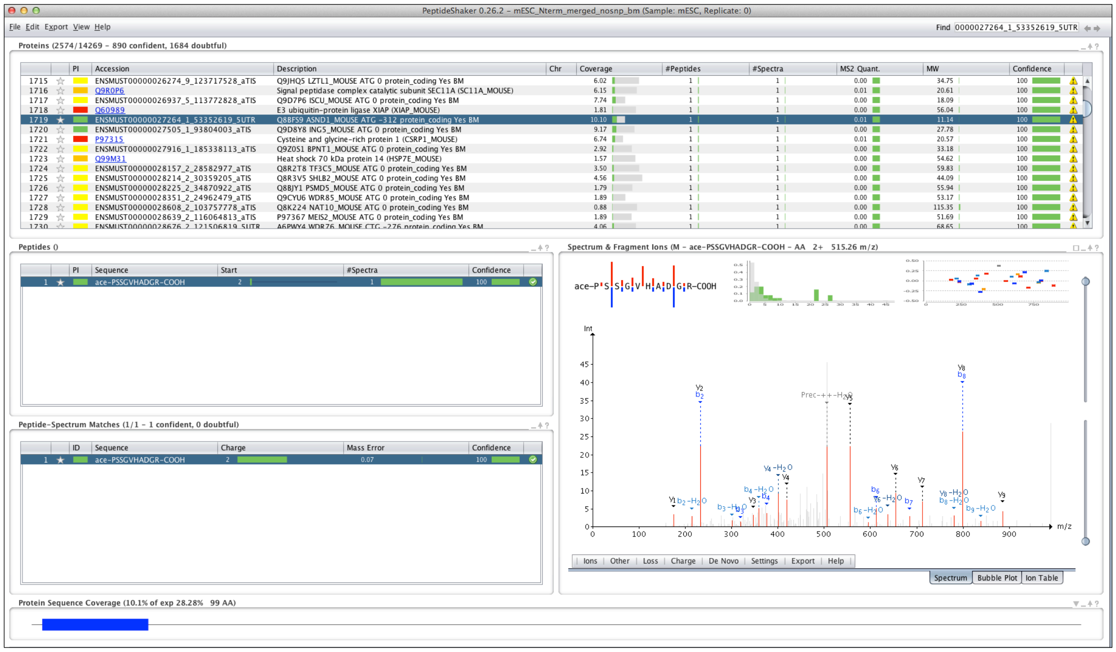

Ensembl info:

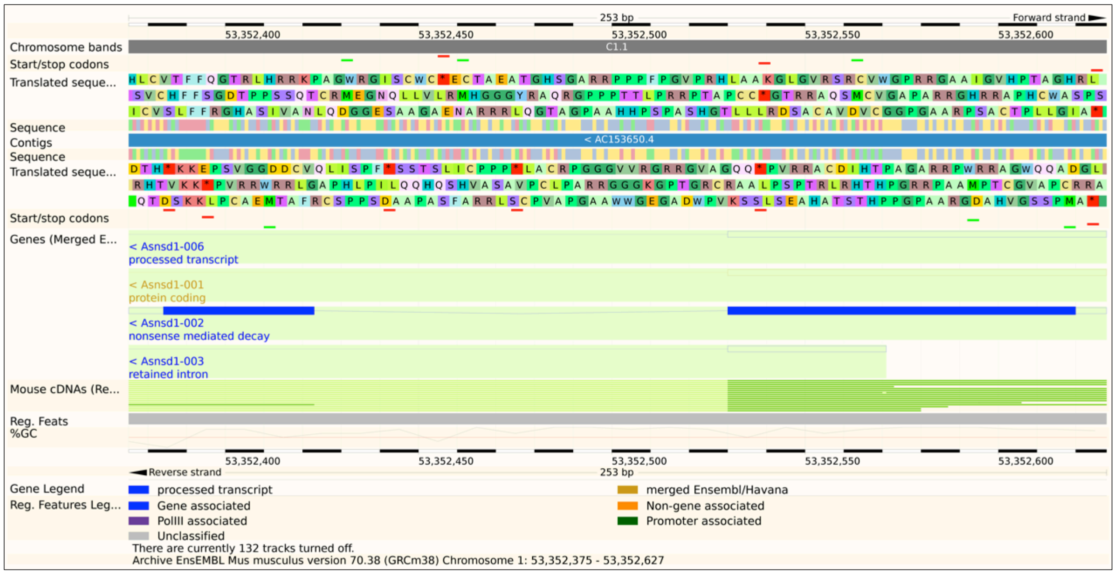

FGENESH info:

FGENESH 2.6 Prediction of potential genes in Mouse genomic DNA
Seq name:
ENSMUSG00000026095|ENSMUST00000027264;ENSMUST00000144660;ENSMUST0000[illegible]
Length of sequence: 31200
Number of predicted genes 4: in +chain 2, in -chain 2.
Number of predicted exons 9: in +chain 7, in -chain 2.
Positions of predicted genes and exons: Variant 1 from 1, Score:108.725366

CDSf CDSi CDSl CDSo PolA TSS

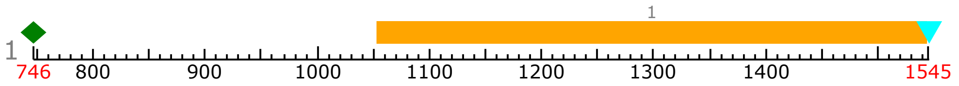

1	-		PolA	746			0.93				
1	-	1	CDSo	1052	-	1543	14.72	1052	-	1543	492
1	-		TSS	1545			-10.59				

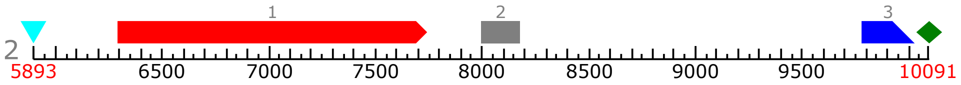

2	+		TSS	5893			-8.99				
2	+	1	CDSf	6287	-	7738	74.07	6287	-	7738	1452
2	+	2	CDSi	7992	-	8173	23.34	7992	-	8171	180
2	+	3	CDSl	9774	-	10023	5.12	9775	-	10023	249
2	+		PolA	10091			0.93				

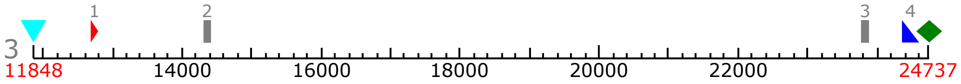

3	+		TSS	11848			-10.49				
3	+	1	CDSf	12672	-	12780	12.59	12672	-	12779	108
3	+	2	CDSi	14295	-	14399	8.12	14297	-	14398	102
3	+	3	CDSi	23760	-	23869	-0.94	23762	-	23869	108
3	+	4	CDSl	24345	-	24593	4.72	24345	-	24593	249
3	+		PolA	24737			0.93				

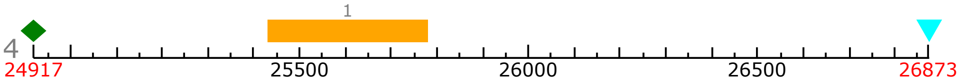

4	-		PolA	24917			0.93				
4	-	1	CDSo	25428	-	25778	12.58	25428	-	25778	351
4	-		TSS	26873			-5.49				

Predicted protein(s):

>FGENESH:[mRNA] 1 1 exon (s) 1052 - 1543 492 bp, chain -
ATGCAGATCTCAGGTGCAGAGGATACCATAGAAAACATTGAAACAACAGTCAAGGAAAAT
GCAAATTGCAAAAAGCTCCTAACCCAAAATATCCAGGAAATCCAGGACACAATGACAAGA
CCAAATCTAAGGATAACAGGTATAGAAGAGAGTGAAGATTCCCAACTTAAATGGTCAGTA
AATATCCTCAACAAAATTAAACAAGAAAACTTTCCTAACCTAAAGAATTTGATGCCCATG
AACATACAAGAAGCCTACAGAACTCCAAATAAATTGGACCAGAAAAGAAATTCCTCCCAT
CACATAATAATCAAAATACCAAATGCACTAAACAAACAAACAAACAAAAGAATATTAAAA
GCAGTAAGGAAAAAGGGTGAAGTAACATGCAAAGTCATACCTATCAGAATTACACCAGAC
TTCTCAGCAGAGACTATGAAAGCTGGAAGATCCTGGGCAGATGTCATACAGACCCTAAGA
GACCACAAATAG
>FGENESH: 1 1 exon (s) 1052 - 1543 163 aa, chain -
MQISGAEDTIENIETTVKENANCKKLLTQNIQEIQDTMTRPNLRITGIEESEDSQLKWSV
NILNKIKQENFPNLKNLMPMNIQEAYRTPNKLDQKRNSSHHIIIKIPNALNKQTNKRILK
AVRKKGEVTCKVIPIRITPDFSAETMKAGRSWADVIQTLRDHK
>FGENESH:[mRNA] 2 3 exon (s) 6287 - 10023 1884 bp, chain +
ATGTGTGGCATTTGCTGTTCTGTAAGCTTCTCTATTGAACACTTCAGTAAAGAGTTAAAA
GAGGATTTGCTGCATAATCTTAGACGGCGGGGCCCCAACAGCAGCAGGCAGTTGTTAAAA
TCTGCTGTTAACTATCAGTGTTTATTTTCTGGTCATGTTCTTCATTTAAGAGGTGTTTTG
ACTATCCAACCTGTAGAAGATGAACATGGCAATGTGTTCTTATGGAATGGAGAAGTTTTT
AATGGAGTAAAGGTTGAAGCAGAAGATAATGACACCCAGGTTATGTTCAATAGCCTTTCT
GCCTGTAAGAATGAGTCTGAAATTTTGCTGCTCTTCTCTAAAGTGCAAGGTCCATGGTCG
TTTATCTATTATCAGGCCTCTAGCCATCACTTATGGTTTGGTAGGGACTTTTTTGGTCGG
CGTAGCTTGCTTTGGCAGTTTAGTAATCTGGGCAAGAGTTTCTGCCTTTCGTCAGTTGGT
ACCCAGGTATATGGAGTTGCAGACCAGTGGCAAGAAGTTCCAGCATCTGGAATTTTCCAG
ATTGATCTCAATTCTGCTGCTGTTTCCAGATCTGTGATCTTAAAATTATATCCTTGGAGA
TACATTTCTAAGGAGGATATTGCCGAAGAATGTGGTAATGACCTGACTCAGACTCCAGCA
GGATTGCCAGAGTTTGTATCAGTGGTAATAAATGAAGCCAACCTGTACCTCTCAAAACCT
GTCGTTCCCTTAAATAAGAAGCTGCCTGAGAGTCCATTGGAAATCCAATGTAGAAACAGT
TCTAGCACTTCAGGTACAAGAGAGACACTTGAGGTATTTCTTACAGATGAACACACAAAA
AAAATAGTTCAGCAGTTCATTGCCATCCTCAATGTTTCAGTCAAGAGACGCATCTTATGT
TTAGCTAGGGAAGAAAACCTGGCATCAAAGGAAGTTTTAAAAACTTGCAGTTCGAAAGCA
AACATTGCGATCCTGTTTTCTGGAGGTGTTGATTCTATGGTGATTGCAGCCCTTGCTGAT
CGTCATATTCCTTTAGATGAGCCAATTGATCTTCTGAATGTGGCTTTTGTGCCTAAACAA
AAAACAGGGCTACCTATTCCTAACATAGAAAGAAAACAGCAGAACCACCATGAGATCCCT
TCTGAAGAGTCCTCTCAGAGTCCTGCTGCAGATGAGGGGCCAGGTGAGGCTGAGGTACCA
GACCGAGTCACAGGAAAAGCAGGACTAAAGGAACTACAGTCTGTCAACCCTTCTCGAACT
TGGAATTTTGTGGAAATAAATGTTTCTCTTGAAGAACTACAAAAACTAAGAAGAGCTCGA
ATATGTCACTTAGTTCAGCCATTGGACACAGTTCTGGATGATAGCATTGGCTGTGCTGTG
TGGTTTGCTTCTAGAGGAATCGGTTGGTTGGTGACCCAAGATGCTGTGAGATCTTACAAG
AGCAGTGCAAAGGTGATTCTTACTGGGATTGGTGCAGATGAGCAGTTGGCAGGTTATTCC
CGTCATCGTGCCCGCTTTCAGTCTCTTGGCCTAGAAGGACTGAACGAGGAAATAGCAATG
GAATTGGGTCGCATTTCTTCTAGAAACCTTGGTCGTGATGACAGAGTTATTGGTGATCAT
GGAAAGGAAGCAAGATTTCCTTTCCTGGATGAAAATGTTGTGTCTTTCCTAAATTCTCTG
CCAGTTTGGGAAAAGGTAGACCTCACTCTGCCCCGTGGAGTTGGTGAGAAGCTTATTTTA
CGCCTTGCAGCTATGGAACTTGGTCTCCCAGCCTCTGCCCTTCTGCCAAAACGAGCCATA
CAATTTGGATCTAGAATTGCAAAACTGGAAAAATCTAATGAGAAGGCATCTGATAAGTGT
GGAAGGCTCCAAATCCTACCTTAG
>FGENESH: 2 3 exon (s) 6287 - 10023 627 aa, chain +

```
MCGICCSVSFSIEHFSKELKEDLLHNLRRRGPNSSRQLLKSAVNYQCLFSGHVLHLRGVL
TIQPVEDEHGNVFLWNGEVFNGVKVEAEDNDTQVMFNSLSACKNESEILLLFSKVQGPWS
FIYYQASSHHLWFGRDFFGRRSLLWQFSNLGKSFCLSSVGTQVYGVADQWQEVPASGIFQ
IDLNSAAVSRSVILKLYPWRYISKEDIAEECGNDLTQTPAGLPEFVSVVINEANLYLSKP
VVPLNKKLPESPLEIQCRNSSSTSGTRETLEVFLTDEHTKKIVQQFIAILNVSVKRRILC
LAREENLASKEVLKTCSSKANIAILFSGGVDSMVIAALADRHIPLDEPIDLLNVAFVPKQ
KTGLPIPNIERKQQNHHEIPSEESSQSPAADEGPGEAEVPDRVTGKAGLKELQSVNPSRT
WNFVEINVSLEELQKLRRARICHLVQPLDTVLDDSIGCAVWFASRGIGWLVTQDAVRSYK
SSAKVILTGIGADEQLAGYSRHRARFQSLGLEGLNEEIAMELGRISSRNLGRDDRVIGDH
GKEARFPFLDENVVSFLNSLPVWEKVDLTLPRGVGEKLILRLAAMELGLPASALLPKRAI
QFGSRIAKLEKSNEKASDKCGRLQILP
>FGENESH:[mRNA] 3 4 exon (s) 12672 - 24593 573 bp, chain +
ATGCACATTCCCGGCCTAAGGCGTAACCTGCATGATGGAGGCCCTAGGACAGCTTTAACT
GGCTCAGGGGTTTCCCAGGAGTTCGAACCAACTTTAGCCCTCAGCACAGCAAGTCCTGGA
TACACCATCACATCAGAAAAGGAAGACATGGATCTAAAGTCACTTCTCATGATGATGATT
GATGACTTTAAGAAGGAAGTACAGGAAACCAGAGGTAATTTAATAGCTAGCCTGGCTCAC
TCGAGGGCTGGGATTCCAGAGGCTTTTTTCTCACTGGGAGCAATCCAGCAGCTCTGCCAC
CACCTGTACTCAGGAAGCGAAGAGGTTCGCACAGCATGTTCCTGTGCCCTTTGCTACCTC
ACTTACAATGCACATGCTTTCCGACTTCTGTTAACTGAGTGTAGCAATAAGCCGAACCAA
TTCCTGCGCATAACAAATAACATCAGTAAAGATGCAAAGATCAATCCTGCGTTCCTAAAG
GAGTTTCAACTGCAGCAAAGGATGAGACTTCCATCCTTAAGGTACTATGCCTTTATGGCC
TTGTTGGACATCAATGGGAGGAGAGGCCCTTAG
>FGENESH: 3 4 exon (s) 12672 - 24593 190 aa, chain +
MHIPGLRRNLHDGGPRTALTGSGVSQEFEPTLALSTASPGYTITSEKEDMDLKSLLMMMI
DDFKKEVQETRGNLIASLAHSRAGIPEAFFSLGAIQQLCHHLYSGSEEVRTACSCALCYL
TYNAHAFRLLLTECSNKPNQFLRITNNISKDAKINPAFLKEFQLQQRMRLPSLRYYAFMA
LLDINGRRGP
>FGENESH:[mRNA] 4 1 exon (s) 25428 - 25778 351 bp, chain -
ATGGGAAGAGAGAAGGAGAAAATGGAAGAGGGAGAGGATGCAGAGGAGAAAGAAGAAGAG
GAGGAGGAGGAAGAAGAAGAAGAAGAGGAGGAGGAGGAGGAAGAAGAAGAGGAGGAGGAG
GAGGGAGAGGTAGAAGAGGAGGAGGAGGTAGAGAGAGGGAGGAGAAGGAGAAGAGGAGGA
GGAGGAAGAAGAAGAAGAGGAGGGGGAGGGAGAAGGGGAAGAGGAGGAGGAAGAGGAGGA
GGGGGAGGAGGAAGAAGAGGAGGAAGCAGAAGAAGGAGGAGGAGGGAGAAGGAGAAGAGG
AGGAGAAGGAAGCGGAAGAAGGAGGAGGAGGAGGGAGGAGAAGAAGAGTAG
>FGENESH: 4 1 exon (s) 25428 - 25778 116 aa, chain -
MGREKEKMEEGEDAEEKEEEEEEEEEEEEEEEEEEEEEEEEGEVEEEEEVERGRRRRRGG
GGRRRRGGGGRRGRGGGRGGGGGGRRGGSRRRRRREKEKRRRRKRKKEEEEGGEEE
```

The spectral matching properties are good but FGenesh could not predict a proteoform including this 5’ uORF sequence. Still this could point to a 5’uORF.

- Detected uORF translation products from HCT116 shotgun experiment:

ENST00000369092_10_121347728_5UTR

PeptideShaker info:

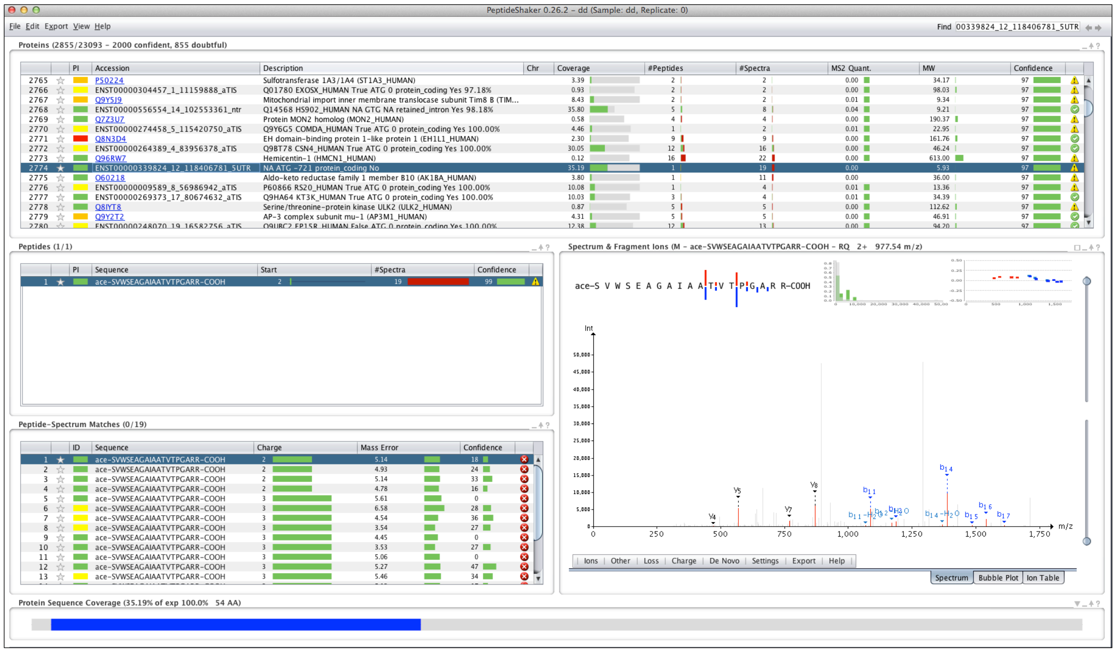

Due to the high peptide mass error (8Da) and rather low PSM confidence, this identification was not retained.

ENST00000339824_12_118406781_5UTR

PeptideShaker info:

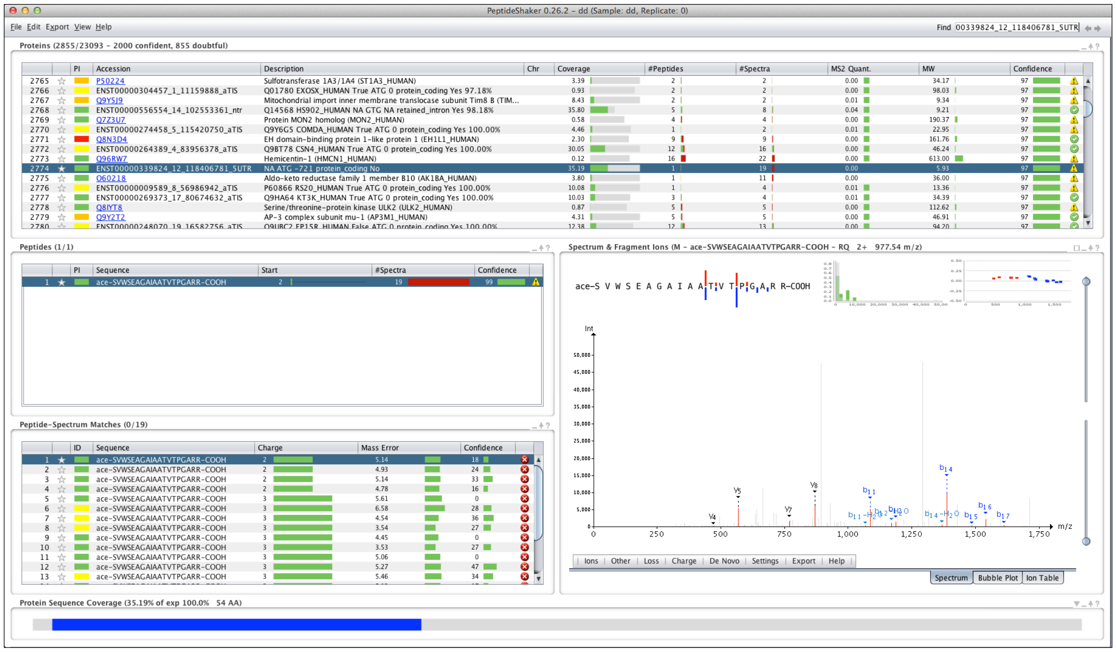

Due to the rather high peptide mass error (5Da) and too low PSM confidence, this identification was not retained.

Supplementary File S2

Readme file for manual installation of the PROTEOFORMER script-based tool.
See attached text file **Suppl_File_S2_README_cmd.txt**

Supplementary File S3

Readme file for the implementation of the PROTEOFORMER approach within a Galaxy instance.
See attached text file **Suppl_File_S3_README_Galaxy.txt**

Supplementary Table S1

General overview of peptide and protein identifications. (a) List of all 3 772 mouse protein products identified in mESC cell lysates. (b) List of all 2 853 human protein products identified in HCT116 WT (wild type) cell lysates. (c) List of all 1 589 mouse protein N-terminal peptides (start = 1 or 2, Arg-C type, Nterm Ac or $^{13}C_2D_3Ac$) identified in mESC cell lysates. (d) List of all 1 312 human protein N-terminal peptides (start = 1 or 2, Arg-C type, Nterm Ac or $^{13}C_2D_3Ac$) identified in HCT116 WT (wild type) cell lysates.
See attached Excel spreadsheet **Suppl_Table_S1.xlsx**

Supplementary Table S2

Mapping statistics. The table provides the read alignment statistics by sample and treatment (CHX or LTM/HARR), throughout the different steps of the mapping using the STAR and TopHat transcriptome mappers.
See attached Excel spreadsheet **Suppl_Table_S2.xlsx**

References

1. Menschaert, G., Van Criekinge, W., Notelaers, T., Koch, A., Crappe, J., Gevaert, K. and Van Damme, P. (2013) Deep proteome coverage based on ribosome profiling aids mass spectrometry-based protein and peptide discovery and provides evidence of alternative translation products and near-cognate translation initiation events. *Molecular & cellular proteomics : MCP*, **12**, 1780-1790.
2. Ong, S.E., Blagoev, B., Kratchmarova, I., Kristensen, D.B., Steen, H., Pandey, A. and Mann, M. (2002) Stable isotope labeling by amino acids in cell culture, SILAC, as a simple and accurate approach to expression proteomics. *Molecular & cellular proteomics : MCP*, **1**, 376-386.
3. Guo, H., Ingolia, N.T., Weissman, J.S. and Bartel, D.P. (2010) Mammalian microRNAs predominantly act to decrease target mRNA levels. *Nature*, **466**, 835-840.
4. Staes, A., Van Damme, P., Helsens, K., Demol, H., Vandekerckhove, J. and Gevaert, K. (2008) Improved recovery of proteome-informative, protein N-terminal peptides by combined fractional diagonal chromatography (COFRADIC). *Proteomics*, **8**, 1362-1370.
5. Staes, A., Impens, F., Van Damme, P., Ruttens, B., Goethals, M., Demol, H., Timmerman, E., Vandekerckhove, J. and Gevaert, K. (2011) Selecting protein N-terminal peptides by combined fractional diagonal chromatography. *Nature protocols*, **6**, 1130-1141.
6. Van Damme, P., Van Damme, J., Demol, H., Staes, A., Vandekerckhove, J. and Gevaert, K. (2009) A review of COFRADIC techniques targeting protein N-terminal acetylation. *BMC proceedings*, **3 Suppl 6**, S6.
7. Vaudel, M., Barsnes, H., Berven, F.S., Sickmann, A. and Martens, L. (2011) SearchGUI: An open-source graphical user interface for simultaneous OMSSA and X!Tandem searches. *Proteomics*, **11**, 996-999.
8. Ingolia, N.T., Lareau, L.F. and Weissman, J.S. (2011) Ribosome profiling of mouse embryonic stem cells reveals the complexity and dynamics of mammalian proteomes. *Cell*, **147**, 789-802.
9. Ishihama, Y., Oda, Y., Tabata, T., Sato, T., Nagasu, T., Rappsilber, J. and Mann, M. (2005) Exponentially modified protein abundance index (emPAI) for estimation of absolute protein amount in proteomics by the number of sequenced peptides per protein. *Molecular & cellular proteomics : MCP*, **4**, 1265-1272.
10. Paoletti, A.C., Parmely, T.J., Tomomori-Sato, C., Sato, S., Zhu, D., Conaway, R.C., Conaway, J.W., Florens, L. and Washburn, M.P. (2006) Quantitative proteomic analysis of distinct mammalian Mediator complexes using normalized spectral abundance factors. *Proceedings of the National Academy of Sciences of the United States of America*, **103**, 18928-18933.

11. Barsnes, H., Vizcaino, J.A., Eidhammer, I. and Martens, L. (2009) PRIDE Converter: making proteomics data-sharing easy. *Nature biotechnology*, **27**, 598-599.
12. Martens, L., Hermjakob, H., Jones, P., Adamski, M., Taylor, C., States, D., Gevaert, K., Vandekerckhove, J. and Apweiler, R. (2005) PRIDE: the proteomics identifications database. *Proteomics*, **5**, 3537-3545.
13. Edgar, R., Domrachev, M. and Lash, A.E. (2002) Gene Expression Omnibus: NCBI gene expression and hybridization array data repository. *Nucleic acids research*, **30**, 207-210.
14. Barsnes, H., Vaudel, M., Colaert, N., Helsens, K., Sickmann, A., Berven, F.S. and Martens, L. (2011) compomics-utilities: an open-source Java library for computational proteomics. *BMC bioinformatics*, **12**, 70.
15. Flicek, P., Ahmed, I., Amode, M.R., Barrell, D., Beal, K., Brent, S., Carvalho-Silva, D., Clapham, P., Coates, G., Fairley, S. *et al.* (2013) Ensembl 2013. *Nucleic acids research*, **41**, D48-55.
16. Solovyev, V., Kosarev, P., Seledsov, I. and Vorobyev, D. (2006) Automatic annotation of eukaryotic genes, pseudogenes and promoters. *Genome biology*, **7 Suppl 1**, S10 11-12.
17. Sievers, F., Wilm, A., Dineen, D., Gibson, T.J., Karplus, K., Li, W., Lopez, R., McWilliam, H., Remmert, M., Soding, J. *et al.* (2011) Fast, scalable generation of high-quality protein multiple sequence alignments using Clustal Omega. *Molecular systems biology*, **7**, 539.
